# The Methodological Quality and Intervention Fidelity of Randomised Controlled Trials Evaluating Social Skills Group Programs in Autistic Adolescents: A Systematic Review and Meta-analysis

**DOI:** 10.1007/s10803-023-05893-z

**Published:** 2023-01-21

**Authors:** Bahareh Afsharnejad, Melissa H. Black, Marita Falkmer, Sven Bölte, Sonya Girdler

**Affiliations:** 1 School of Allied Health, Curtin University, Kent Street, Bentley, Perth, WA 6102 Australia; 2 Curtin Autism Research Group (CARG), Curtin University, Perth, WA Australia; 3 School of Education and Communication, CHILD, Swedish Institute for Disability Research, Jönköping University, Jönköping, Sweden; 4 Division of Neuropsychiatry, Department of Women’s and Children’s Health, Center of Neurodevelopmental Disorders (KIND), Centre for Psychiatry Research, Karolinska Institutet & Child and Adolescent Psychiatry, Stockholm Health Care Services, Stockholm County Council, Stockholm, Sweden; 5 School of Allied Health, University of Western Australia, Perth, WA Australia

**Keywords:** Autism, Adolescents, Social skills, Randomised controlled trial, Systematic review

## Abstract

A systematic review and meta-analysis were utilised to explore the methodological quality, program fidelity, and efficacy of social skills group programs (SSGPs) aiming to support autistic adolescents in navigating their everyday social worlds. The study evaluated the methodological quality and theoretical fidelity of studies, with a random effect meta-analysis conducted to summarise the overall efficacy of SSGP and its effect on social communication and interaction, behavioural/emotional challenges, adaptive functioning, and autism characteristics. Although findings from the 18 identified studies indicated an adjusted medium overall effect with these programs successfully supporting autistic adolescents’ socialisation needs (g = 0. 60, *p* < 0.001), most studies demonstrated medium to low program fidelity despite their good methodological quality. Given the significant heterogeneity of SSGPs and variations in the design and measurement frameworks of efficacy studies, understanding the generalisability of the findings of this research is unclear.

## Introduction

Adolescence is a period of rapid development during which youth continue to define themselves in relation to their social environment, form their self-esteem, and shape their self-concept (DuBois et al., 1998; Smetana et al., 2006). During adolescence, youth more commonly engage in activities with their peers and less with their families, seeking opportunities to expand their social networks and develop social skills (Smetana et al., 2006). Like their neurotypical (NT) peers, most autistic^1^ youth desire peer friendships and wish to engage in social activities (Bauminger & Kasari, 2003). Despite these aspirations, autistic adolescents engage less frequently in social activities than their NT counterparts (Askari et al., 2015). Although the factors impacting the social interactions of autistic adolescents are complex, their experiences are likely, at least in part, impacted by the different social communication and interaction styles inherent to a diagnosis of autism (American Psychiatric Association [APA], 2013; Bottema Beutel et al., 2021). Restricted engagement in social activities due to social communication differences limits autistic adolescents’ opportunities to gain experience and practice social skills (Askari et al., 2015; Majnemer et al., 2015; Smetana et al., 2006), increasing their risk of social isolation and withdrawal (Bauminger & Shulman, 2003). In the long term, participation restrictions may negatively impact autistic adolescents’ mental health and adult outcomes (Ratcliff et al., 2018), hampering independent living, employment, and further education opportunities (Howlin & Magiati, 2017).

### Social Skills Group Programs

To date, intervention development has focussed on designing specialised (Radley et al., 2020; Tseng et al., 2020) and generic (Naveed et al., 2019) psychosocial programs aiming to support autistic individuals in navigating the neurotypical world (Lerner et al., 2012). Social skills group programs (SSGPs) are most frequently delivered to school-aged autistic youth with average or above-average general cognitive abilities (IQ > 70). SSGPs vary in their theoretical underpinnings, content, teaching strategies, delivery modes, and intensity. Despite this variability, SSGPs commonly focus on supporting participants to develop their interpersonal skills, social knowledge, and the social performance necessary to achieve their social goals within a neurotypical world (Wolstencroft et al., 2018). Overall, SSGPs are most frequently delivered by one to three trainers in weekly 60 to 90-min sessions (12 to 16 sessions) to a small group of autistic youth (two to six individuals). Ultimately these programs aim to support participants in generalising their practised or newly acquired skills to their everyday social contexts (Reichow et al., 2010; Wolstencroft et al., 2018).

SSGPs can be delivered in a structured or semi-structured format, employing either explicit didactic, implicit performance-based teaching strategies or both (Wolstencroft et al., 2018). Given that the success of explicit programs relies on translating knowledge into behaviour, the outcomes of programs employing these teaching approaches largely depend on participants’ level of motivation and concentration (Guivarch et al., 2017). In contrast, implicit teaching approaches focus on delivering opportunities for participants to develop their social skills during immersive activities, focusing on changing behaviour rather than the overt teaching of skills.

#### Current Evidence

Recent decades have seen an increase in published studies evaluating the efficacy of SSGPs targeting the social skills of autistic youth. Across efficacy studies, there is considerable variability in the components underpinning these programs and the measurement frameworks employed in evaluating their efficacy. The need to understand the efficacy of these approaches more broadly has led to the publication of several systematic reviews in this field.

Recent systematic reviews synthesising the literature for SSGPs, targeting autistic youth evaluated via randomised controlled trial (RCT) design, suggest a modest treatment efficacy in the areas of social knowledge and performance (Gates et al., 2017) and a reduction in autism characteristics (Wolstencroft et al., 2018). However, these findings should be interpreted within the context that they are almost exclusively underpinned by reports from informants other than autistic youth themselves (Gates et al., 2017; Monahan et al., 2021) and the broad age range of participants in the included studies (ranging from 5 to 25 years), with only one review noting the potential moderating effect of age (developmental stage) on study outcomes (Gates et al., 2017). Notably, research on SSGPs is dominated by samples of male school-age autistic children with an IQ > 70 and of European ancestry (Jonsson et al., 2016). Further, this body of research has largely disregarded the opinions of autistic individuals in developing the content or format of these interventions (Monahan et al., 2021). Collectively, these issues call into question both the external and social validity of SSGPs.

### The Current Review

Although previous reviews have contributed significantly to our understanding of the efficacy of SSGPs in increasing the social knowledge and social skills of autistic youth in navigating the neurotypical social world, some limitations remain. Firstly, despite SSGPs demonstrating some efficacy in increasing autistic youth’s knowledge of the social skills commonly utilised in the neurotypical world, there has been little consideration of program fidelity (PF), that is, whether the program is administered as initially intended (Gates et al., 2017; Tseng et al., 2020). Judging the *true* efficacy of SSGPs when PF is unclear or unreported is virtually impossible, given that other unaccounted-for factors may influence the intervention’s efficacy (Borrelli, 2011). To date, no review has systematically explored the degree to which SSGPs were delivered as initially intended (Borrelli, 2011). Further, previous systematic reviews only included RCTs evaluating the efficacy of SSGP compared to the waitlist or no-treatment control groups (Gates et al., 2017; Wolstencroft et al., 2018). It remains unclear whether the observed effects of these programs resulted from participants' exposure to a structured, supportive group context or the SSGPs alone (Gates et al., 2017). Evidence further suggests that SSGPs are likely more efficacious for autistic adolescents than children (Choque Olsson et al., 2017). To advance understanding of the efficacy of SSGPs in autistic adolescents, this systematic review firstly assessed the methodological quality and PF of studies evaluating the efficacy of SSGPs in improving autistic adolescents’ (aged 12 to 17 years) socialisation success within a neurotypical context via an RCT design. Subsequently, a meta-analysis of outcomes categorised as social communication and interaction skills, behavioural/emotional challenges, adaptive functioning, and autism characteristics investigated the impact of SSGPs on autistic adolescents in these specific domains. This review also included studies employing active controls as a means of controlling for exposure to the social context in judging the efficacy of these programs.

## Method

This systematic review and meta-analysis was guided by the Preferred Reporting Items for Systematic Reviews and Meta-Analysis (PRISMA) statement (Liberati et al., 2009). This review was registered with PROSPERO (*identifier* CRD42020213178) on 24 October 2020.

### Eligibility Criteria

Studies evaluating a SSGP to improve the socialisation success of autistic adolescents within a neurotypical context were included in this review. Although this review focused on programs targeting autistic adolescents, studies employing samples with a broader age range (including younger children) were also included. Studies examining the efficacy of school-delivered SSGPs were excluded, constraining the heterogeneity of programs and focusing on SSGPs delivered in clinical settings. The hallmark features of school-delivered SSGP, occurring in youth’s everyday social context facilitated by classroom teachers familiar with participants, make these programs inherently different from those delivered within clinical settings (Kasari et al., 2016). SSGPs primarily focussing on parent or family outcomes, in preference to improving the socialisation success of autistic adolescents within a neurotypical context, were also excluded (Table 1).

**Table 1:** Inclusion and exclusion criteria and search strategy

Eligibility criteria and search strategy	
Inclusion criteria	
Program	Social Skills Group programs (SSGP) targeting the socialisation success of autistic adolescents within a neurotypical context
Participant	Autistic adolescents diagnosed with ASD under DSM-5 or its previously recognised terms under DSM-IV (Autism, Autistic disorder, Asperger’s Syndrome, Pervasive developmental disorder-not otherwise specified). Although the preferred age range was 12 to 17 years, programs employing samples with a broader or narrower age range were also tolerated
Design	A randomised controlled trial focusing on adolescent outcomes
Documents	Scholarly articles
Exclusion criteria	School-based programs; Studies not targeting the social communication and interaction skills of autistic adolescents as one of the primary objectives or focussing on parent or family outcomes
Where	Title, abstract, and keywords
Search strategy	1. ‘Social’ 2. ‘Program’ OR ‘treatment’ OR ‘training’ OR ‘therapy’ 3. ‘Teen*’ OR ‘adolescen*’ OR ‘youth’ OR ‘Juvenile’ 4. ‘Randomised controlled trial’ OR ‘RCT’ OR ‘Randomised’ 5. ‘Pervasive developmental disorder’ OR ‘autis*’ OR ‘Asperger’ OR ‘ASD’ OR ‘PDD’ 6. 1 AND 2 AND 3 AND 4 AND 5 7. Limit to 2008 to 2018/update: limit to 2018 to 2020

*ASD* autism spectrum disorder, *DSM-IV* Diagnostic and Statistical Manual of Mental Disorders-Fourth Edition, *DSM-5* Diagnostic and Statistical Manual of Mental Disorders-Fifth Edition, *PDD* pervasive developmental disorder, *RCT* randomised controlled trial, *SSGP* social skills group program

### Information Sources and Search Strategy

Six electronic databases (CINAHL, Medline, ProQuest, PsycINFO, Clarivate, and Scopus) were searched for scholarly articles published in English from 2008 until December 2018 and later updated to November 2020, describing SSGPs aiming to improve the social communication and interaction skills of autistic adolescents. Title, abstract, and keyword searches were undertaken in each database. The main keyword search terms were grouped into five categories (‘autism’, ‘social’, ‘program’, ‘adolescents’, and ‘RCT’). They were then combined with related terms via Boolean operators (Table 1) and tailored to each database. The reference lists of the identified articles were searched for further manuscripts meeting the eligibility criteria. Studies identified via study registrations and personal communication were also included in this review.

### Study Selection

All citations were imported into Endnote referencing manager, and duplicates were removed. Articles were screened at the title and abstract level for eligibility against the inclusion criteria. The full texts of candidate articles were subsequently retrieved and assessed for eligibility by two reviewers (BA, MB).

### Data Extraction

A data extraction form designed for the purposes of this review was developed guided by the Cochran Handbook for systematic reviews (Higgins & Green, 2011). Extracted data included the study design and randomisation process, sample size, inclusion/exclusion criteria, recruitment strategy, facilitators and setting characteristics, incentives, program/comparison group characteristics, assessment time points, outcome measures (primary and secondary), collection of fidelity and adverse events related to the SSGP, type of analysis and a summary of results.

### Assessment of Methodological Quality and Risk of Bias

Two reviewers trained and experienced in conducting systematic reviews (BA, MB) independently rated the quality of all included articles using the Standard Quality Assessment Criteria for Quantitative Studies for quantitative and qualitative studies (Kmet et al., 2004). This 14-item checklist assesses the methodological quality of articles regarding (a) clarity of the aim and design, (b) sample size calculation, (c) control group selection, (d) randomisation process, (e) blinding to group allocation (participants, investigators collecting data, or both), (f) the robustness of outcome measures, (g) analytic methods including some estimates of variance, (h) the sufficiency of reported results, and (i) relevant conclusions. Single items were scored on a scale of 0 (not achieved) to 2 (criteria met), with total proportional scores calculated (by dividing the total raw score by the possible maximal score of the relevant items) and converted to percentage scores, enabling categorisation of articles according to their methodological quality [> 80% strong, 70–80% good, 50–70% adequate and < 50% limited methodological quality (Lee et al., 2008)]. The reviewers compared the ratings for all included studies, with discrepancies discussed until consensus was reached.

### The Procedure for Assessing Program Fidelity

The Treatment Fidelity Assessment and Implementation Plan was used to examine and summarise the extent to which each study delivered its program as initially planned (Borrelli, 2011). This 30-item checklist assessed strategies used in each study to ensure adherence concerning: program design (*k* = 7 items reflecting adherence to the underlying theoretical framework of the program); training of providers (*k* = 7 items assessing the standardisation of the training process); delivery (*k* = 9 items quantifying the level of rigour employed in assessing fidelity assessment during the trial); receipt of the program (*k* = 5 items describing the participants receiving the program) and; enactment of program skills (*k* = 2 items covering the assessment monitoring and improvement in participants’ performance of taught skills both within and outside the program context). Each checklist item is scored on a dichotomous scale of 1 (present) or 0 (not reported). These checklist items were subsequently used to calculate five subscale scores and one overall score (Bellg et al., 2004). Possible scores range from 0 to 1, with proportional scores of > 0.80 indicating high levels of PF (Borrelli et al., 2005). This measure has shown good reliability and validity, with programs with higher PF scores (total proportional scores) found to be more efficacious (Borrelli et al., 2005; Johnson-Kozlow et al., 2008). Two reviewers (BA, MB) independently rated all included studies, with discrepancies resolved via discussion.

### Meta-analysis

Six meta-analyses were performed according to the meta-analytic procedures suggested by Liberati et al. (2009). Two explored the effect of SSGPs on all outcome measures used within the studies immediately after completion of the program and at 3-month follow-up. The remaining four investigated the effect of SSGP on four outcome categories used across these studies as described below (social outcomes, behavioural/emotional challenges, adaptive functioning, and autism characteristics). Data and the script required to replicate the process is available at https://osf.io/n93pu/.

#### Term Parameters—Outcome Categories

The first author grouped the outcomes into four overarching categories to address the heterogeneity of outcome measures employed to assess the efficacy of SSGPs, facilitating synthesis across studies (Table 2). All authors then reviewed these categories, discussing differences in opinion. This process resulted in four agreed categories: social outcomes, behavioural/emotional challenges, adaptive functioning, and autism characteristics. Social outcomes or social communication and interaction skills defined measures assessing autistic adolescents’ social knowledge or social behaviour (when socialising within a neurotypical context). Behavioural/emotional challenges measures included measures aiming to assess autistic adolescents’ internalising and externalising behaviours, including their emotional states and emotion regulation (Achenbach & Edelbrock, 1978). Adaptive functioning was defined as multidimensional measures capturing autistic adolescents’ ability to effectively and independently cope with everyday demands (Harrison & Boney, 2002). Autism characteristics defined measures employed to diagnose autism (APA, 1994, 2013) or quantify its characteristics.

**Table 2:** Outcome measure categories based on the outcomes used in the included studies

Category	Outcome measure
Social outcomes (social interaction and communication skills)	Test of Adolescent Social Skills Knowledge _ Revised (TASSK), Friendship Qualities Scale (FQS), Mutual Engagement, NEPSY-II, Positive facial expression, Quality of Play Questionnaire (QPQ), Quality of Socialisation Questionnaire (QSQ), Social inquiries, Social interaction observation system, (SIOS), Social Motivation and Competencies Scale (SMCS), Social preference, Social Skills Improvement Scale (SSIS), Social Skills Rating System (SSRS), Sociometrist nomination, The Contextual Assessment of Social Skills (CASS), The Peer Interaction Paradigm (PIP)
Behavioural/emotional challenges	Child and Adolescent Symptom Inventory-4 ASD Anxiety scale (CASI-ANX), Child Behaviour Checklist (CBCL), Child Depression Inventory (CDI), Children in Stress (CiS), Depressioninventar fur Kinder- und Jugendliche (DIKJ), Emotion Quotient (EQ), Loneliness and Social Dissatisfaction Questionnaire (LSDQ), Paediatric Anxiety Rating Scale (PARS), Perceived Stress Scale (PSS), Revised UCLA Loneliness Scale, Social Interaction Anxiety Scale (SIAS), Strength and Difficulties Questionnaire (SDQ), The State Anxiety Inventory
Adaptive functioning	Adaptive Behaviour Assessment System II (ABAS), Developmental Disabilities Children’s Global Assessment (DD-CGASevaluating their efficacy is provided), OSU Autism Clinical Global Impression – Severity (CGI-S), Vineland Adaptive Behaviour Scale (VABS)
Autism characteristics	Asperger Syndrome Diagnostic Scale (ASDS), Autism Diagnostic Observation Schedule (ADOS), Social Communication Questionnaire (SCQ), Social Responsiveness Scale (SRS)

#### Statistical Analyses

The findings of studies conducted by Schohl et al. (2014) and Van Hecke et al. (2015) were found to be from an overlapping sample. In line with the process described by Gates et al. (2017), only the study with the more complete data set was included in the meta-analysis (Schohl et al., 2014).

Some studies only reported outcome measures demonstrating significant change in a measure’s total score, subscales, or both. Studies presenting only results for subscales were excluded from the analysis, decreasing heterogeneity across included studies and improving the internal validity of the meta-analysis. Estimates of effect size with a bias correction (Hedges’ g) were calculated by dividing the mean difference of the outcome measures for both SSGPs and control groups from baseline to post-test/follow-up by the pooled standard deviation of study groups at baseline (Morris, 2008). F values or t values were used to calculate the effect sizes in studies where the means and standard deviations were not reported. (Borenstein et al., 2009).

Separate random-effects meta-analyses (as outlined under meta-analysis) were performed using RStudio Version 4.2.1 (RStudio Team, 2015) and its available packages (metaphor, compute.es, and MAd; Del Re, 2013, 2015; Del Re & Hoyt, 2018). Effect sizes and variances within individual studies were aggregated for the meta-analysis process to enable a more precise estimate of the studies’ effect and account for any possible variance within and between the studies (Borenstein et al., 2009). A coefficient value of 0.5 was set for each category, as the correlations between outcome measures within each category were not readily available (Borenstein et al., 2009). Statistical significance was set at *p* < 0.05, with an effect size (Hedges’ g) of < 0.2, indicating a small, 0.2–0.5 a medium and > 0.8 a large SSGP effect (Fritz et al., 2012). Heterogeneity among effects was assessed using a restricted maximum-likelihood estimator for Tau^2^ and Chi-Square statistics with an inconsistency score (*I*^*2*^) of 25% demonstrating low, 50% moderate, and 75% high levels of heterogeneity (Higgins et al., 2003). An influence diagnostic assessment (e.g., Baujat plots) investigated how individual studies had affected heterogeneity (Enea & Plaia, 2014). Meta-regression moderator analysis was performed when 10 and more studies with high heterogeneity were included in the meta-analysis. Moderator analysis assessed whether methodological quality, PF, age group, gender, and exposure to the SSGP (as calculated in minutes) had influenced the effect sizes.

#### Publication Bias

Funnel plots and Egger’s test were used to estimate the possibility of publication bias by plotting the observed effect size against standard errors on the y-axis (Egger et al., 1997). A further sensitivity test was performed as a visual inspection of the funnel plots’ asymmetry alone cannot account for publication bias (Bartoš et al., 2020). If significant publication bias was present at *α* = 0.01 (Bartoš et al., 2020), a robust bias correction was performed to adjust the findings using JASP (https://jasp-stats.org). JASP is a free program developed to support conducting classical and Bayesian forms of meta-analysis.

## Results

### Search Results

Electronic database searches identified 3,337 articles. Upon removing duplicates, 1880 articles’ titles and abstracts were reviewed, with the full text of twenty-three articles subsequently evaluated for eligibility. Seven articles did not meet the inclusion criteria for reasons including (a) not employing an RCT design (*k* = 2), (b) not targeting social communication and interaction skills (*k* = 2), (c) targeting children younger than the age range of this review (*k* = 1), (4) focusing on parent and family outcomes (*k* = 1), and (5) not being a peer-reviewed journal article (*k* = 1). A review of the reference lists of eligible articles and trial registries identified two further studies, resulting in 18 articles being included in the narrative synthesis.

**Fig. 1:**
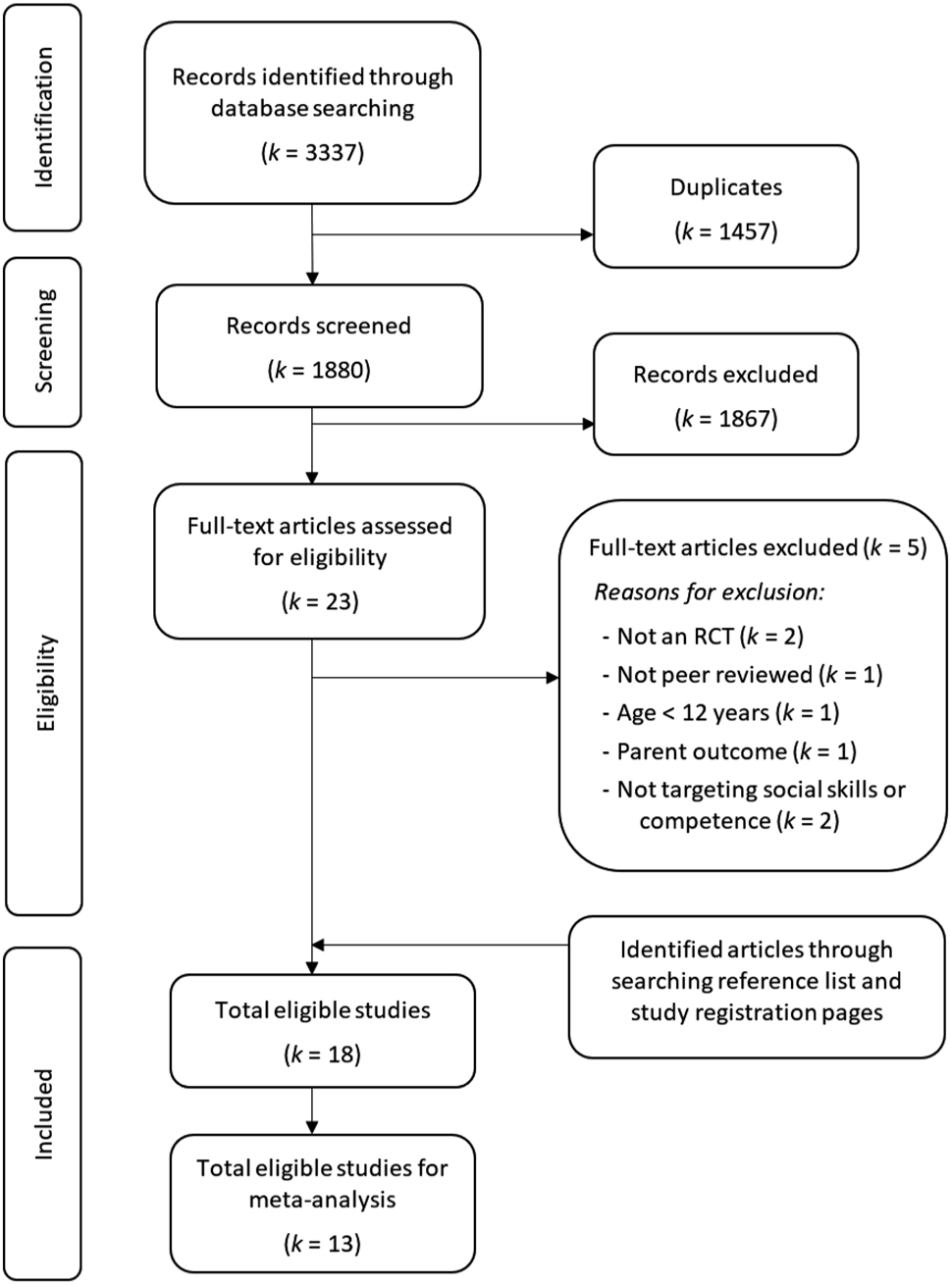
Selection of studies of social skills group training for autistic youth

The eligibility of the eighteen articles included in the systematic review was assessed for inclusion in the meta-analysis. When manuscripts presented insufficient data to support meta-analysis (i.e., presented results for subscales only), corresponding authors were contacted (n = 7). Authors from two studies responded and provided the requested data. Five studies were therefore excluded from the meta-analysis (Corbett et al., 2019; Matthews et al., 2018, 2020; Van Hecke et al., 2015; Vernon et al., 2018). The selection process is presented in Fig. 1.

### Narrative Synthesis

Overall, the included studies (*k* = 18) evaluated seven unique manualised SSGPs delivered to autistic youth with IQ > 70, including (a) Program for the Education and Enrichment of Relationship Skills (PEERS®; *k* = 8; Laugeson et al., 2009; Matthews et al., 2018; Matthews et al., 2020; Rabin et al., 2018; Schohl et al., 2014; Shum et al., 2019; Van Hecke et al., 2015; Yoo et al., 2014), (b) KONTAKT® (*k* = 3; Choque Olsson et al., 2017; Jonsson et al., 2018), (c) SENSE Theatre® (*k* = 2; Corbett et al., 2016; Corbett et al., 2019), (d) Social Tools and Rules for Teens (START; *k* = 2; Ko et al., 2019; Vernon et al., 2018), (e) Multimodal Anxiety and Social Skills Program (MASSI; *k* = 1; White et al., 2013), (f) Sociodramatic Affective Relational Intervention (SDARI; *k* = 1; Lerner & Mikami, 2012), and (g) SOSTA-FRA (*k* = 1; Freitag et al., 2016). The majority of the studies were conducted in the United States of America (USA; 65%), with the remainder undertaken in Australia (Afsharnejad et al., 2021a, 2021b), China (Shum et al., 2019), Germany (Freitag et al., 2016), Israel (Rabin et al., 2018), Korea (Yoo et al., 2014) and Sweden (Choque Olsson et al., 2017; Jonsson et al., 2018).

These seven SSGPs employed varied teaching strategies, including structured (k = 4; 57%), semi-structured (k = 2; Ko et al., 2019; Vernon et al., 2018) and unstructured, performance-based approaches (k = 1; Lerner & Mikami, 2012). The majority of SSGPs were delivered to autistic youth in weekly 90-min sessions. The number of sessions varied across SSGPs, with the shortest program delivered over four sessions (Lerner & Mikami, 2012). The longest SSGP, KONTAKT®, was delivered over twenty-four sessions (Jonsson et al., 2018), being an extension of a shorter 12-session (Choque Olsson et al., 2017) and medium 16-session variants (Afsharnejad et al., 2021a, 2021b). Sessions were commonly delivered to small groups of between 3 to 10 participants. Although most studies targeted adolescents aged 11–17 years, the efficacy of KONTAKT®, SENSE Theatre, and SOSTA-FRA was evaluated with samples including both children and adolescents. All SSGPs were reportedly led by therapists/clinicians, with several involving trained and supervised coaches and NT peers. Parents were engaged in providing feedback and educated on ways to support their child during the program, with this role extending to coaching in the PEERS® and the MASSI programs. The KONTAKT®, SOSTA-FRA, START, and MASSI programs incorporated individualised goal setting or tailored planning, with the first three developing these in collaboration with autistic youth. KONTAKT®, PEERS®, SOSTA-FRA, and START, incorporated individually tailored homework assignments to support the generalisation of learnt skills to everyday contexts. Uniquely, the long (Jonsson et al., 2018) and medium (Afsharnejad et al., 2021a, 2021b) variants of KONTAKT® incorporated components supporting the in-vivo assessment of learnt skills within the sessions (participants leading a session) and the generalisation of skills to a community context (an excursion to a café). In assessing the efficacy of SSGPs programs, four studies employed active control groups (Lerner & Mikami, 2012; Matthews et al., 2018, 2020) or attempted to control for the effects of exposing autistic adolescents to a supportive social context (Afsharnejad et al., 2021a, 2021b). The remaining studies assessed the efficacy of SSGPs compared to treatment as usual or waitlist control. Only half of the included studies reported the setting where the SSGP was delivered, which included meeting rooms at community centres, clinical outpatient units, university settings and at school (after school hours). The measurement frameworks and informants used in establishing efficacy varied. A more detailed description of the included SSGPs and the studies evaluating their efficacy is provided in Appendix 1 (Tables 4, 5, 6).

### Methodological Quality Analysis

Of the 18 studies, eight (41%) detailed the flow of participants through their studies in CONSORT diagrams (Afsharnejad et al., 2021a, 2021b; Choque Olsson et al., 2017; Jonsson et al., 2018; Schohl et al., 2014; Shum et al., 2019; Vernon et al., 2018; White et al., 2013). Overall, included studies demonstrated good to strong methodological quality (i.e., scores of > 75% as assessed via the Kmet Checklist). Methodological limitations mainly included (a) small sample sizes, (b) failure to conduct a blind assessment of outcomes, (c) a lack of allocation concealment, and (d) failure to describe randomisation processes or conduct intent-to-treat analysis (Accessible via https://osf.io/n93pu/).

### Program Fidelity Analysis

All 18 studies but one (Rabin et al., 2018) reported assessing PF, with studies employing various methods, including assessing video recordings of randomly chosen sessions using a fidelity checklist (Afsharnejad et al., 2021a, 2021b; Choque Olsson et al., 2017; Jonsson et al., 2018; White et al., 2013), observing a session assessing fidelity either in reference to the program manual (Shum et al., 2019; Yoo et al., 2014) or via a checklist. The included studies demonstrated overall PF scores ranging from 0.33 to 0.90 (*M* = 0.52, *SD* = 0.15), with 17% (*k* = 3) demonstrating a strong overall fidelity (Afsharnejad et al., 2021a, 2021b; Choque Olsson et al., 2017; Jonsson et al., 2018). An overview of the PF scores is available via https://osf.io/n93pu/.

#### Design

The seven items of this category assessed the degree to which studies reflected the stated theoretical constructs and mechanisms of the program. All included studies demonstrated a good program design score, ranging from 0.57 to 0.92 (*M* = 0.79, *SD* = 0.10), with 47% (*k* = 10) demonstrating a strong fidelity on this criterion (Afsharnejad et al., 2021a, 2021b; Choque Olsson et al., 2017; Freitag et al., 2016; Jonsson et al., 2018; Lerner & Mikami, 2012; Matthews et al., 2018, 2020; Schohl et al., 2014; White et al., 2013; Yoo et al., 2014). Fidelity was negatively affected by a lack of information in relation to contingency planning for managing implementation setbacks (such as drawing on reserve trainers) and providing insufficient detail regarding the program’s underlying theoretical constructs (Corbett et al., 2016, 2019; Ko et al., 2019; Laugeson et al., 2009; Shum et al., 2019; Van Hecke et al., 2015; Vernon et al., 2018) or facilitators’ credentials (Corbett et al., 2016, 2019; Lerner & Mikami, 2012; Rabin et al., 2018).

#### Providers

The seven items in this category assessed the degree to which studies provided standardised training to trainers/coaches. This category received the lowest fidelity score, with scores ranging from 0.14 to 0.71 (*M* = 0.36, *SD* = 0.19). Common fidelity limitations included failing to clearly describe the training materials provided to therapists, coaches, or peers (Freitag et al., 2016; Laugeson et al., 2009; Lerner & Mikami, 2012; Matthews et al., 2020; Rabin et al., 2018; Shum et al., 2019; Van Hecke et al., 2015; White et al., 2013), or approaches to supervision (Freitag et al., 2016; Rabin et al., 2018; Van Hecke et al., 2015). Six studies described assessing trainers’ readiness to deliver the SSGP before commencing (Afsharnejad et al., 2021a, 2021b; Choque Olsson et al., 2017; Corbett et al., 2019; Jonsson et al., 2018; White et al., 2013; Yoo et al., 2014) Three studies described their training as standardised (Afsharnejad et al., 2021a, 2021b; Choque Olsson et al., 2017; Jonsson et al., 2018). Eligibility (Corbett et al., 2019; Matthews et al., 2018), fitness to deliver the program (Corbett et al., 2019) and individualisation of the training process (Matthews et al., 2018) were reported in studies drawing on neurotypical co-leader peers. However, these studies provided limited details in relation to the training of therapists leading the groups.

#### Delivery

The nine items of this category assessed the degree to which studies were executed as outlined in their RCT Protocols. Delivery scores ranged from 0.11 to 0.88 (*M* = 0.51, *SD* = 0.22), with six studies (33%) demonstrating strong fidelity in this category (Afsharnejad et al., 2021a, 2021b; Choque Olsson et al., 2017; Corbett et al., 2016, 2019; Freitag et al., 2016; Jonsson et al., 2018; White et al., 2013). Across the included studies common limitations included (a) failure to specify fidelity scores a priori (e.g., adhere to delivering > 80% of components; Corbett et al., 2016; Corbett et al., 2019; White et al., 2013), (b) omitting a description of the strategies employed in delivering the programs (e.g., reinforcement, prompting; Ko et al., 2019; Laugeson et al., 2009; Lerner & Mikami, 2012; Matthews et al., 2018, 2020; Rabin et al., 2018; Schohl et al., 2014; Shum et al., 2019; Van Hecke et al., 2015; Vernon et al., 2018; White et al., 2013; Yoo et al., 2014), (c) failure to specify if scripts were used in delivering SSGP curriculum (Corbett et al., 2016, 2019; Ko et al., 2019; Vernon et al., 2018; White et al., 2013) and (d) not monitoring adverse events or nonspecific program effects (Corbett et al., 2016, 2019; Ko et al., 2019; Laugeson et al., 2009; Lerner & Mikami, 2012; Matthews et al., 2018, 2020; Rabin et al., 2018; Schohl et al., 2014; Shum et al., 2019; Van Hecke et al., 2015; Vernon et al., 2018; White et al., 2013; Yoo et al., 2014). Only the study comparing the efficacy of the KONTAKT® to an active control group (Afsharnejad et al., 2021a, 2021b) described the strategies employed to mitigate potential contamination threat between the study arms (contact amongst SSGP and control group participants).

#### Receipt of Program

The sum of the five items, assessing whether participants understood and acquired the skills covered in the SSGPs, demonstrated high scores ranging from 0.4 to 1.0 (*M* = 0.73, *SD* = 0.19). Twelve studies (67%) achieved strong fidelity (Afsharnejad et al., 2021a, 2021b; Choque Olsson et al., 2017; Corbett et al., 2016, 2019; Freitag et al., 2016; Jonsson et al., 2018; Ko et al., 2019; Laugeson et al., 2009; Lerner & Mikami, 2012; Matthews et al., 2018; Rabin et al., 2018; Shum et al., 2019; Vernon et al., 2018; Yoo et al., 2014). Fidelity scores were negatively impacted by the failure to report consideration of (a) cultural factors (e.g., assessing cross-cultural acceptability of the program; Corbett et al., 2016, 2019; Ko et al., 2019; Laugeson et al., 2009; Lerner & Mikami, 2012; Matthews et al., 2018, 2020; Schohl et al., 2014; Van Hecke et al., 2015; Vernon et al., 2018; White et al., 2013), (b) participants enactment of learnt skills (e.g., via homework assignments; Corbett et al., 2016, 2019; Lerner & Mikami, 2012; Schohl et al., 2014), (c) comprehension of session content (e.g., reviewing the session at the end; Corbett et al., 2016, 2019; White et al., 2013), or (d) the use of applied strategies during sessions to enhance comprehension (e.g., providing visual aids, workbooks or written session agenda; Matthews et al., 2020; Rabin et al., 2018; Schohl et al., 2014; Shum et al., 2019; Van Hecke et al., 2015; Vernon et al., 2018; White et al., 2013; Yoo et al., 2014).

#### Enactment of Program Skills

The two items assessing the enacting of program skills pertained to trainers’ assessment of participants' skills either within or outside the SSGPs sessions (Borrelli, 2011). Though two studies (11%) demonstrated strong fidelity (*M* = 0.23 [0.00, 1.00], *SD* = 0.34) under this category (Afsharnejad et al., 2021a, 2021b; Jonsson et al., 2018). Scores in this category were negatively impacted by the failure to document the assessment of participants’ performance during group sessions (Corbett et al., 2016, 2019; Freitag et al., 2016; Ko et al., 2019; Laugeson et al., 2009; Matthews et al., 2018, 2020; Rabin et al., 2018; Schohl et al., 2014; Shum et al., 2019; Van Hecke et al., 2015; Vernon et al., 2018; White et al., 2013; Yoo et al., 2014) or other contexts (Freitag et al., 2016; Laugeson et al., 2009; Lerner & Mikami, 2012; Matthews et al., 2018, 2020; Rabin et al., 2018; Schohl et al., 2014; Shum et al., 2019; Van Hecke et al., 2015; Vernon et al., 2018; Yoo et al., 2014).

### Meta-analysis

#### Analysis of Outcomes from Baseline to Post-test

**Fig. 2:**
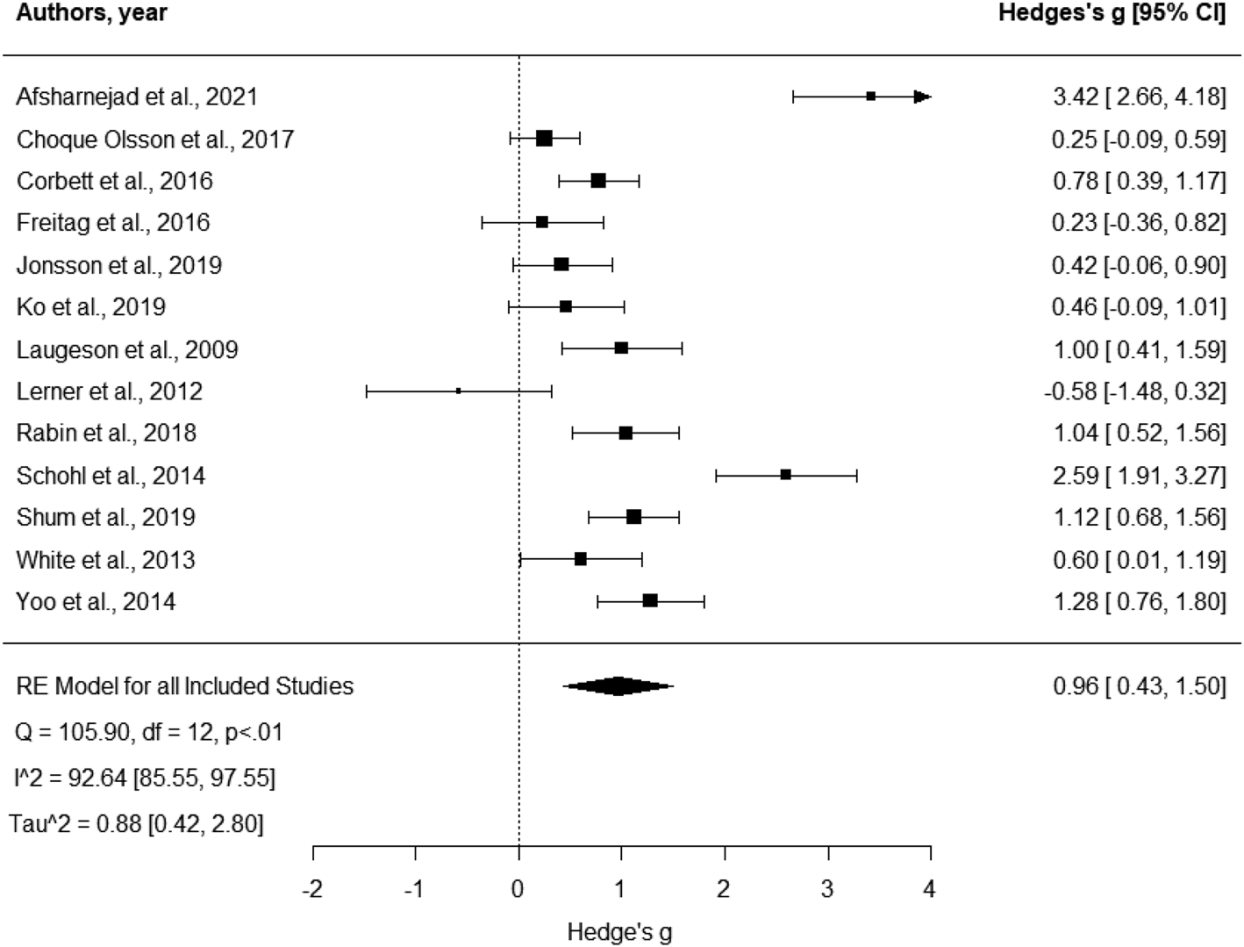
Forest plot comparison of all outcomes. Analysis was based on the aggregated score calculated from the total score of all outcomes used within each study. Positive scores indicate more significant improvement for the intervention group compared to the control group from baseline to post-test

A total of 57 effect sizes (*Mean* = 4.38 per study, *SD* = 2.06, *Median* = 4) from the 13 studies were included in this analysis. These studies overall had strong methodological quality (*M* = 86.31, *SD* = 6.5) and modest PF (*M* = 0.54, *SD* = 0.18). In their RCT, three studies employed usual care, two active controls, and the remaining waitlist controls. Four studies reported pooled results from children and adolescents. According to Hedges’ g, the effect sizes of these studies ranged from -0.58 to 3.42 (Fig. 2 and Table 3).

**Table 3:** Social skills group training effect sizes (Hedges’ g) from baseline to post-test

Meta-analysis	k	Total *N*	Q	Hedges’ g	z	Ʈ^b^	I^b^ (%)
All outcomes from T1 to T2	13	967	144.49***	^a^0.96*** ^b^0.60***	0.52	0.88	92.38
Social outcomes from T1 to T2	9	412	156.81***	1.91**	2.56	4.87	87.66
Behavioural/emotional challenges from T1 to T2	8	790	18.14**	− 0.14	− 1.16	0.07	65.96
Autism characteristics from T1 to T2	8	782	49.5***	− 0.10	− 0.38	0.51	89.16
All outcomes from T1 to T3	6	711	122.24***	1.43	1.54	4.98	99.31

*ASD* autism spectrum disorder, *CI* confidence interval, T1 baseline, T2 posttest, T3 follow-up

^a^Unadjusted

^b^Adjusted

**p < 0.01; ***p < 0.001

The meta-analysis revealed an aggregated large overall effect for the efficacy of SSGPs for improving autistic adolescents’ outcomes from baseline to post-test (Hedges’ g = 0.96, *p* = 0.001, 95% CI [2.71, 4.13]). There was high heterogeneity observed in the included effect sizes. Egger’s regression test indicated no evidence of small study bias (z = 0.71, *p* > 0.05). No significant moderation effects on quality, PF, gender or age were found (*p* < 0.5).

**Fig. 3:**
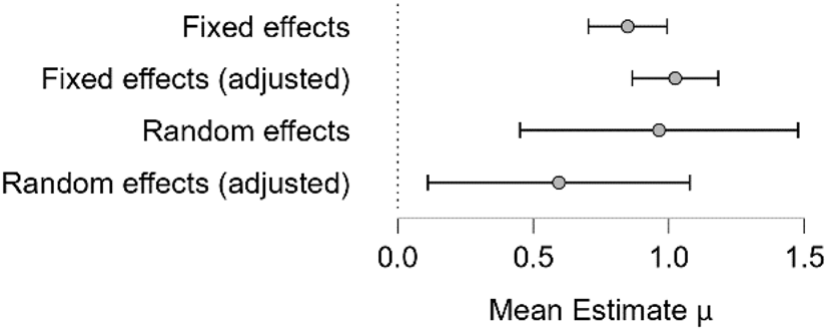
Adjusted and Unadjusted Models. Analysis was based on the Bias Correction model suggested by Bartos et al., 2020, adjusting for publication bias. Positive scores indicate more significant improvement for the intervention group compared to the control group from baseline to post-test

The visual inspection indicated that some studies fell outside the funnel plot. Hence a sensitivity analysis was performed. Findings indicated three studies were influencing the results, indicating a need for a bias correction (Afsharnejad et al., 2021a, 2021b; Lerner et al., 2012; Schohl et al., 2014). The reported effect of SSGP demonstrated a decrease from the unadjusted model to the adjusted one (Fig. 3), suggesting a moderate efficacy (μ = 0.60, *p* < 0.001, 95% CI [0.11, 1.08]).

#### Analysis of Maintenance Effects from Baseline to Follow-Up

**Fig. 4:**
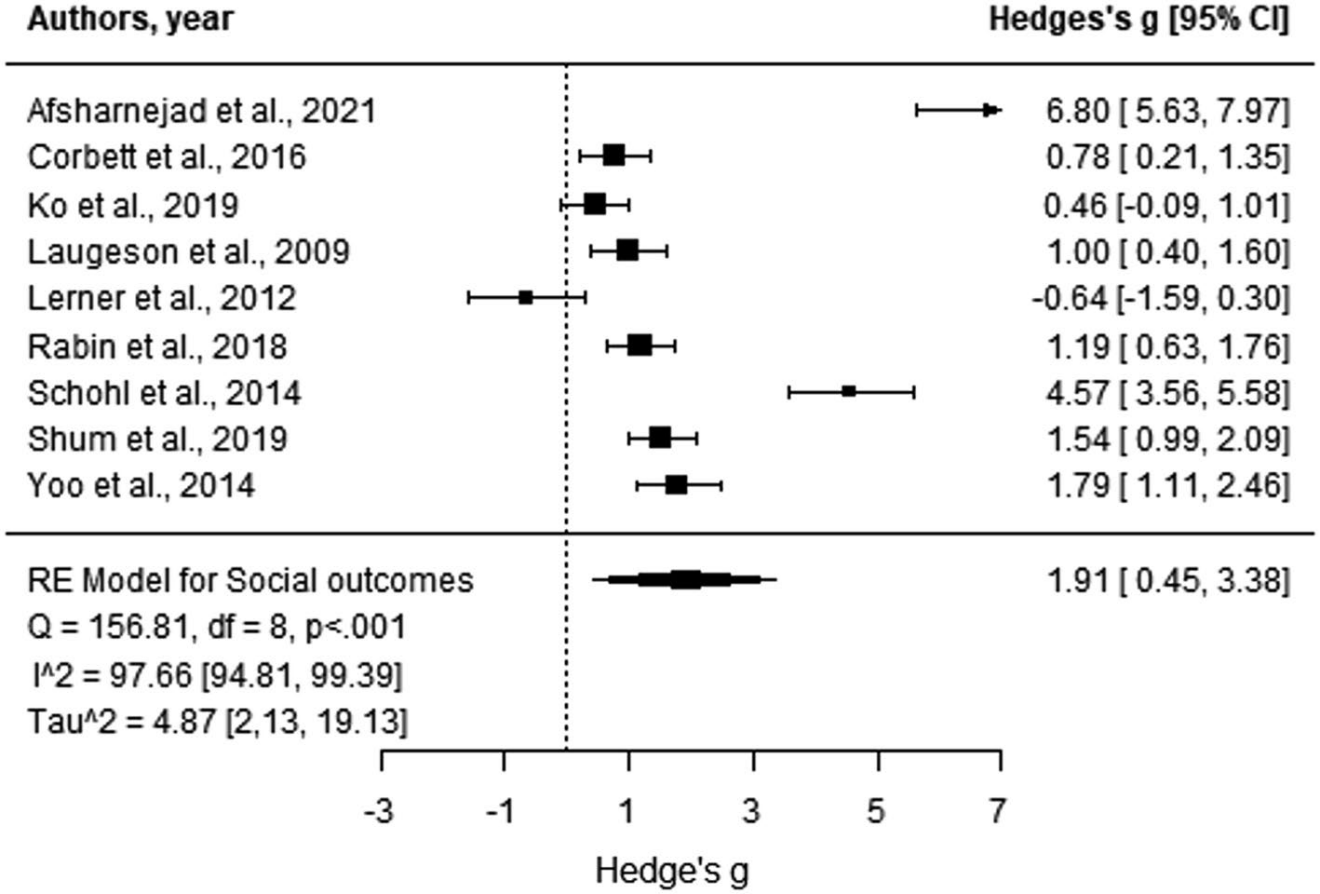
Forest plot comparison of the social outcomes category outcome measures. Analysis was based on the aggregated score calculated from the total score of all outcome measures in the social outcome category. Positive scores indicate more significant improvements for the intervention group compared to the control group from baseline to post-test

Five studies provided data at a follow-up time on all their outcomes. The meta-analysis of maintenance effects resulted in effect sizes ranging from 0.24 to 6.15 (Fig. 4). No significant overall maintenance effect was observed at follow-up. This finding supported the conclusion that across studies, from post-test to follow-up, autistic adolescents failed to sustain the benefits they reported directly following the completion of the SSGP (Hedges’ g = 1.43, *p* = 0.12, 95% CI [− 0.38, 3.23]). Findings indicated heterogeneity between effect sizes across the included studies (*Q* = 122.24, *p* < 0.001, *I*^2^ = 99.31% [98.15, 99.89]).

#### Outcome Categories—Analysis of Outcomes from Baseline to Post-test

##### Social Outcomes

Data from all informants underpinned the assessment of social outcomes across the included studies. However, more than half of the reported findings drew on data obtained via adolescents’ self-reports immediately after completion of the SSGP. Social outcomes assessed included (a) measured improvements in social skills knowledge (Laugesson et al., 2009; ES = [0.36, 3.57]) as assessed by the Test of Adolescent Social Skills Knowledge (Laugeson & Frankel, 2010), (b) social skills (Laugeson et al., 2009: ES = 0.68; Vernon et al., 2018: ES = 0.21) as assessed via the Social Skills Rating System (Gresham & Elliott, 1990), and the Social Skills Improvement Scale (Gresham & Elliott, 2008), (c) friendship quality (Laugeson et al., 2009; ES = 0.48) and skills (Schohl et al., 2014: ES = not reported; Rabin et al., 2018: ES = 0.09) as assessed by the Friendship Qualities Scale (Bukowski et al., 1994) and Quality of Socialisation Questionnaire (Laugeson et al., 2009) and (d) hosted get-togethers (Laugeson et al., 2009; ES = 1.04) as assessed via the Quality of Play Questionnaire (QPQ; Frankel & Mintz, 2008). One study undertook a blind assessment of the primary outcome, adolescents’ progress towards their personally meaningful social goals. The progress was measured via goal attainment scaling (Kiresuk et al., 1994), reporting that SSGP participants made more progress towards their goals than those attending a cooking program (active control; Afsharnejad et al., 2021a, 2021b; ES = 0.35).

Six studies obtained social outcome data via observer reports indicating improvements in youth’s social skills across SSGPs. Studies employing the NEPSY-II (Korkman et al., 2007) measured outcomes via both blinded (Corbett et al., 2016) and unblinded means (Corbett et al., 2019), noting improvements in group play (ES = 0.77) immediately following participation in the SENSE Theatre® SSGP (ES = 0.75), and delayed improvements in participants recall of faces (ES = 0.98), engagement in cooperative play (ES = 0.58), verbal interaction (ES = 0.47) and theory of mind (ES = 0.45). Lerner and Mikami (2012) noted that upon completing SDARI, participants had significant decreases in occasions of negative social interactions (positive: ES =  − 1.17; negative: ES =  − 0.98) as assessed via the Social Interaction Observation System (Bauminger, 2002). One study assessing autistic youth’s social skills via the Contextual Assessment of Social Skills (Ratto et al., 2011) reported that participants were more engaged in social situations and asked more questions after completing PEERS® (Rabin et al., 2018; ES = 0.16). After attending START, autistic youth demonstrated improved social competencies (Vernon et al., 2018) as measured via the Social Motivation and Competencies Scale (Chevallier et al., 2012; ES = 0.29), asked more questions (ES = 0.13) and recognised more positive facial expressions (ES = 0.19; Ko et al., 2019).

Only one study suggested PEERS® was efficacious in improving participants’ social skills (Rabin et al., 2018; ES = 0.30) via data from parent proxy reported Social Skills Improvement Scale. Teacher reports collected via the same measure, however, failed to detect any significant differences between groups.

**Fig. 5:**
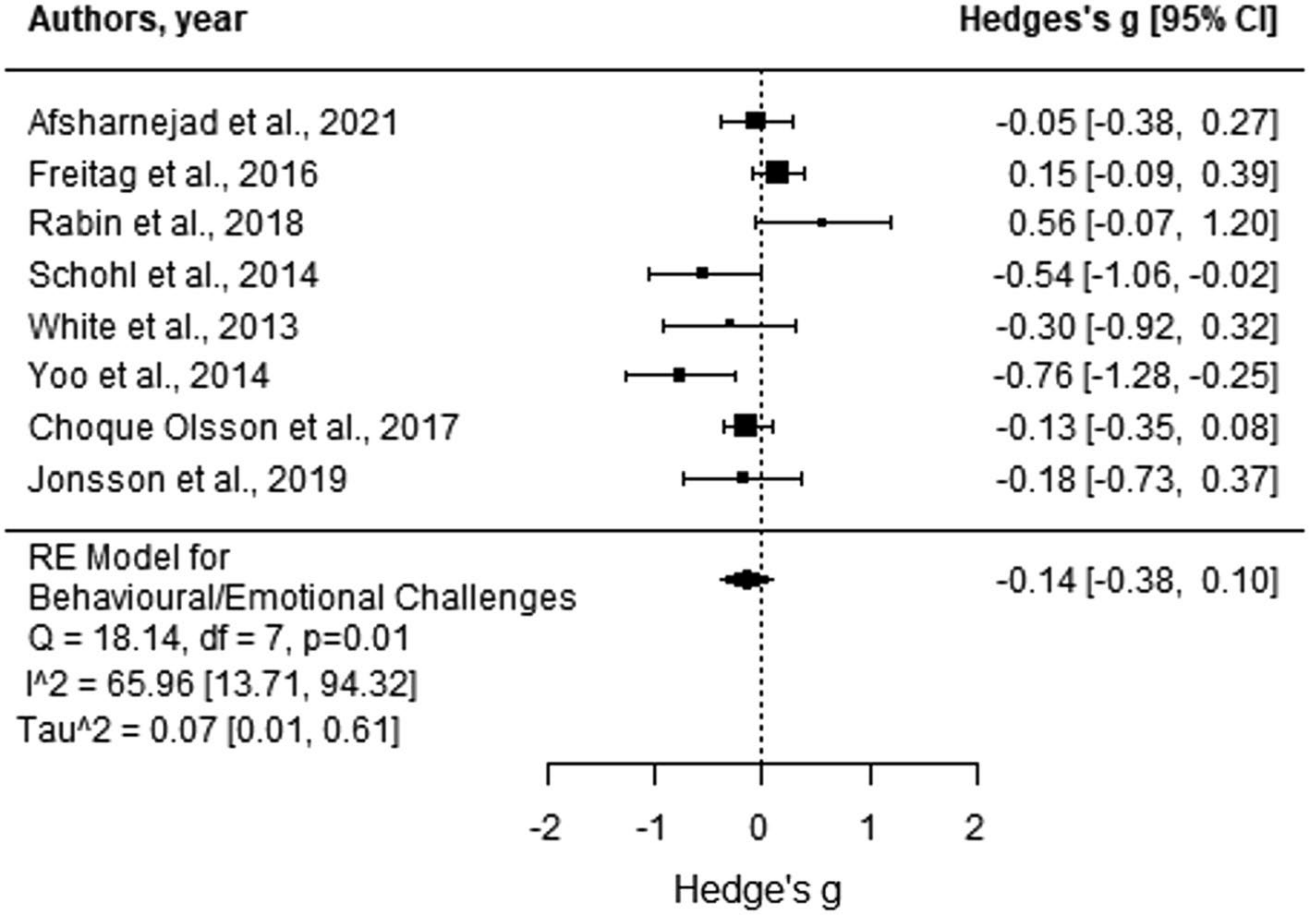
Forest plot comparison of the behavioural/emotional challenges category outcomes. Analysis was based on the aggregated score calculated from the total score of all outcomes in the behavioural/emotional challenges category. Positive scores indicate more significant improvements for the intervention group compared to the control group from baseline to post-test

Based on the meta-analysis, the Hedges’ g effect sizes of the nine studies providing data related to social outcomes ranged from − 0.64 to 6.80 (Fig. 5). Egger’s regression test demonstrated no evidence of publication bias (*p* > 0.05). Findings indicated large efficacy for SSGPs in relation to improving social outcomes from baseline to post-test, showing autistic adolescents attending SSGPs gained significantly more social skills than those in control groups (Hedges’ g = 1.91, *p* = 0.01; 95% CI [0.45, 3.38]). There was significant heterogeneity in effect sizes (Q = 156.81, *p* < 0.001, I^2^ = 97.66% [94.81, 99.39]).

##### Behavioural/Emotional Challenges

Two studies reported reduced social anxiety following participation in SSGPs as assessed via the self-reported Social Interaction Anxiety Scale (Mattick & Clarke, 1998). One study reported this change immediately following the intervention period (Schohl et al., 2014: ES = not reported) and the remaining at 3-months follow-up (Afsharnejad et al., 2021a, 2021b; ES = 0.47). One study utilising the Emotion Quotient (Baron-Cohen & Wheelwright, 2004) reported improvements in emotion regulation (ES = 0.12). A further study assessing participants' prosocial behaviour and psychopathology via parent proxy-reported Strength and Difficulties Questionnaire (Rothenberger et al., 2008) demonstrated a significant improvement in the behavioural and emotional challenges experienced by autistic youth at 3 months follow-up (ES = 0.34; Freitag et al., 2016).

**Fig. 6:**
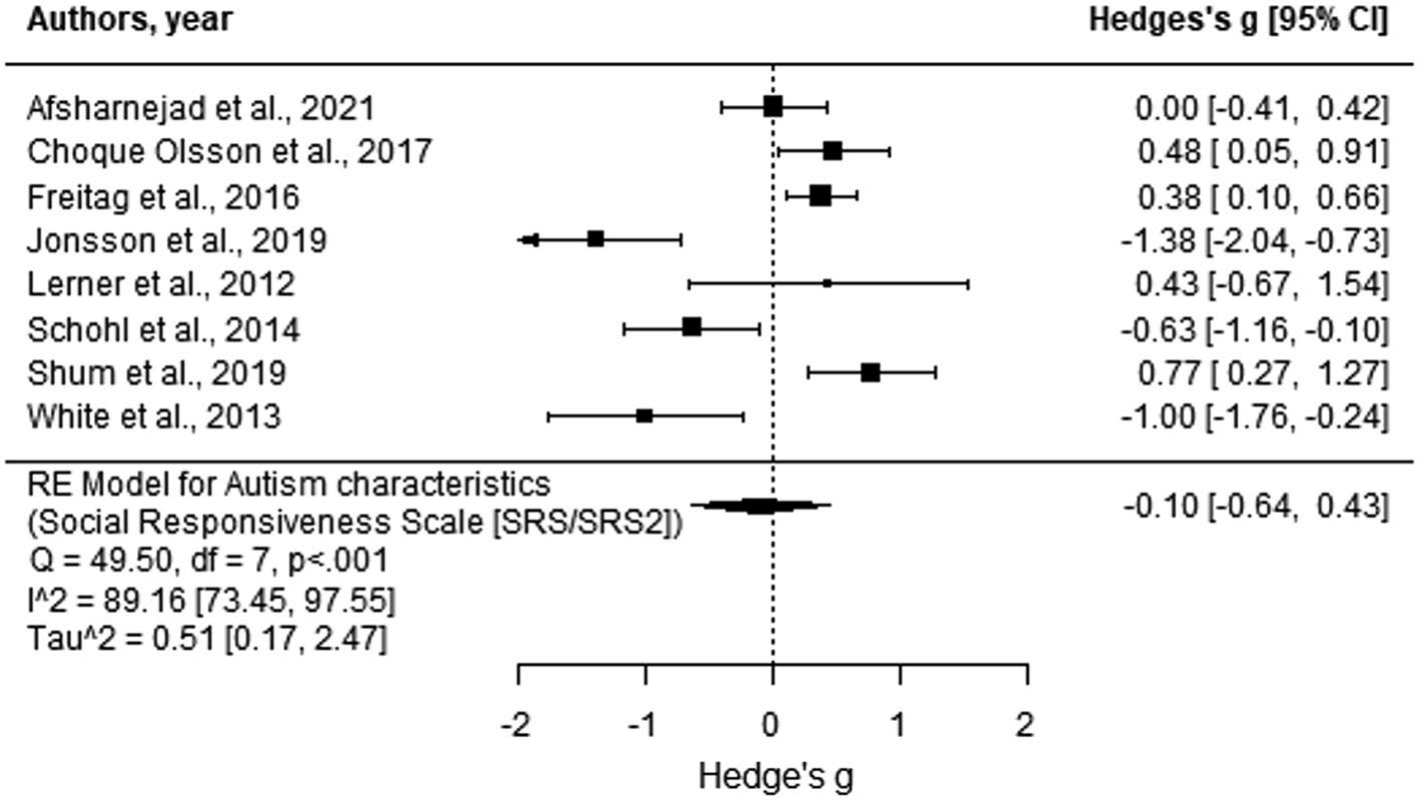
Forest plot comparison of the autism characteristics category outcomes. From the autism symptomology category, the included studies all had only used SRS/SRS-2. As such, the scores are indicative of autistic-like traits. Negative scores indicate more significant improvements for the intervention group compared to the control group from baseline to post-test

The effect sizes of the eight studies contributing data in this category ranged from − 0.76 to 0.56 (Fig. 6), with Egger’s regression test finding no evidence of publication bias (*p* > 0.05). Attending SSGP significantly reduced autistic adolescents’ behavioural and emotional challenges compared to those in control groups from baseline to post-test (Hedges’ g = − 0.14, *p* = 0.25, 95% CI [− 0.38, 0.10]). The analysis suggested no significant heterogeneity of effect sizes (*Q* = 18.14, *p* = 0.01, *I*^2^ = 65.96% [13.71, 94.32]).

##### Autism Characteristics

About half of the included studies (*k* = 9) employed either the SRS (Constantino & Gruber, 2005) or SRS-2 (Constantino & Gruber, 2012) in their measurement frameworks, with six studies denoting it as their primary outcome (Choque Olsson et al., 2017; Freitag et al., 2013; Jonsson et al., 2018; Lerner & Mikami, 2012; White et al., 2013; Yoo et al., 2014). One of these studies reported that parents were blind to group allocation (Lerner & Mikami, 2012). Findings of these studies showed a significant decrease in autistic-liked traits (*p* < 0.05) immediately after attending SSGP ranging from 0.19 to 1.2. Three studies reported this change was sustained at 3-month follow-up (KONTAKT®: ES = [0.33, 0.82]; SOSTA-FRA: ES = 0.34). One study employing a large sample (*n* = 296) reported that female participants demonstrated a greater change in autistic-liked traits than males (Choque Olsson et al., 2017).

**Fig. 7:**
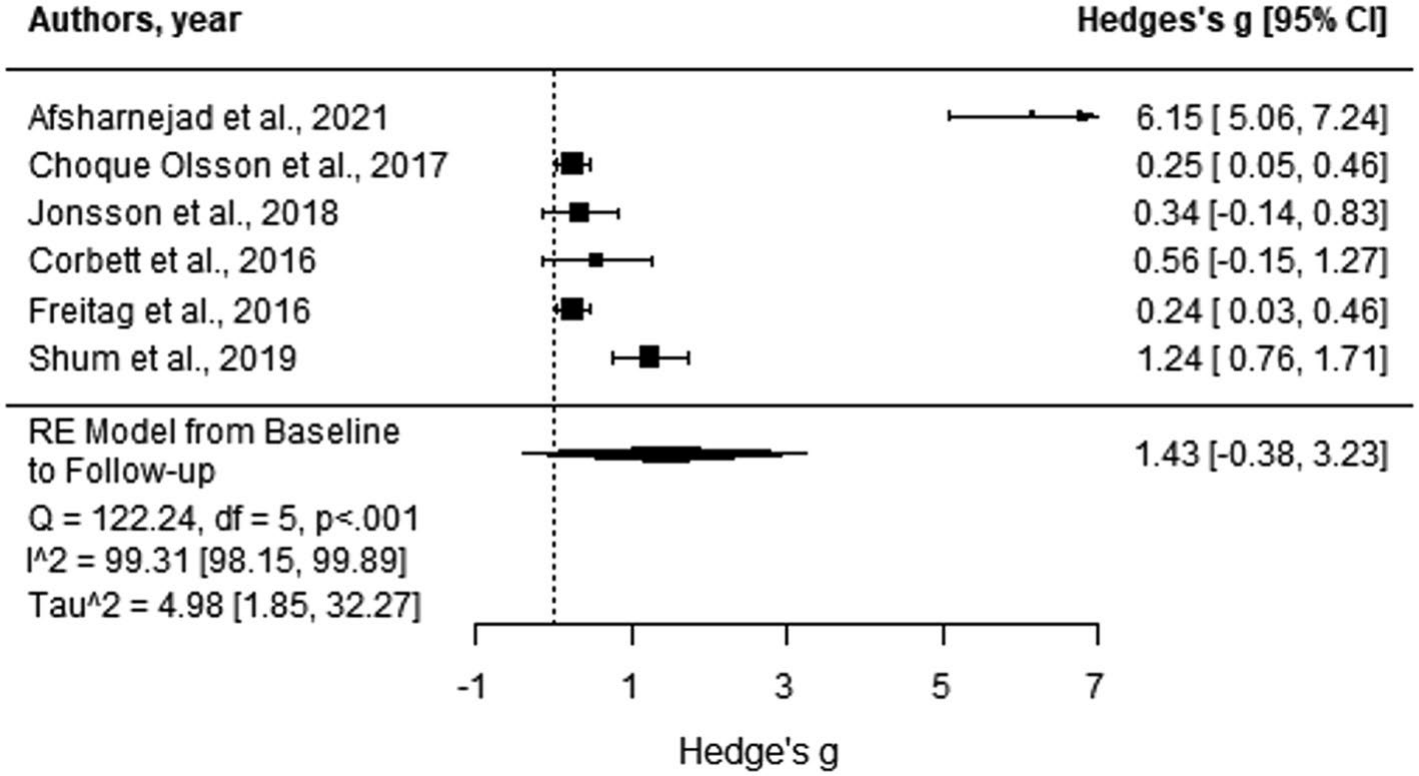
Forest plot comparison of all outcome measures from baseline to follow-up. Analysis was based on the aggregated score calculated from the total score of all outcome measures used within each study. Positive scores indicate more significant improvement for the intervention group

Based on the meta-analysis, the effect sizes of the eight studies contributing to this category (all employing SRS/SRS-2) ranged from − 1.00 to 1.38 (Fig. 7), with Egger’s regression test finding no evidence of publication bias (*p* > 0.05). Findings suggest that overall, attending SSGP did not significantly influence the autistic characteristics of adolescents in comparison to their peers in the control groups, between baseline to post-test (Hedges’ g = − 0.10, *p* = 0.71, 95% CI [− 0.64, 0.43]). The heterogeneity of effect sizes was significant (*Q* = 49.50, *p* < 0.001, *I*^2^ = 89.16% [73.45, 97.55]).

## Discussion

This systematic review was conducted with the express goal of advancing understanding of the methodological quality and PF of studies evaluating the efficacy of SSGPs in samples of autistic adolescents via RCT design. Overall, eighteen studies met the inclusion criteria, evaluating the efficacy of seven distinct manualised programs delivered to small groups of cognitively able autistic adolescents’ (aged 12 to 17 years), aiming to improve participants’ socialisation success within a neurotypical context. Despite the good to strong methodological quality of included studies, the majority demonstrated moderate to low PF. Comparable to previous reviews (Gates et al., 2017), findings of the meta-analysis suggested that SSGPs are moderately effective in supporting autistic youth in navigating the neurotypical world, particularly in developing their social communication and interaction skills.

### Methodological Quality

The present review included studies not previously incorporated in a systematic review. Overall, the included studies demonstrated strong methodological quality in line with previous reviews. The confidence in their findings, though, is constrained by several notable limitations. Notably, in line with previous reviews (Gates et al., 2017; Wolstencroft et al., 2018), the present review identified that most SSGP evaluation studies are underpinned by small samples, likely inflating effect sizes and leading to overestimations of their efficacy (Bukowski et al., 1996). Further, as highlighted in a previous review (Gates et al., 2017), most relevant studies either failed to employ blind assessment of outcome measures or nominated blindly assessed outcomes as the primary outcome. This limitation exposes the current body of evidence to high levels of expectancy bias (Williams et al., 2012), stemming from parents’ expectancy of improvement of their child’s social skills given their investment of time and energy in participating in the trial (McMahon et al., 2013), and both adolescents and researchers’ expectations of improvement (Williams et al., 2012). Few studies in this review specified a primary outcome a priori, as recommended by the CONSORT guideline (Moher et al., 2010), for conducting RCTs limiting comparison across studies.

### Program Fidelity

Uniquely, this review examined the PF of SSGPs delivered to autistic youth. Although the studies included in this review reported assessing the fidelity of their programs via various strategies, few reported the extent to which the delivery of the study aligned with their RCT protocols. This limited reporting and the present review's low to moderate PF scores are of concern (Borrelli et al., 2005; Harden et al., 2015). Low PF scores in an RCT can threaten the reliability and validity of the efficacy findings for SSGPs (Barton & Fettig, 2013; Craig et al., 2008; Harden et al., 2015; Santacroce et al., 2004; Wells et al., 2012). PF is of greater importance when the program is delivered within a community context, where researchers’ ability to control the context is limited, and program facilitators have varied professional backgrounds (Smith et al., 2007; Wells et al., 2012). Due to the increasing number of available SSGPs and limited information provided by the current published reports, future research should focus on assessing the efficacy of SSGPs in community settings when accounting for PF. Considering facilitators’ views or perceptions of SSGPs, and providing systematic training, monitoring and supervision of facilitators, are all strategies likely to enhance the fidelity and translation of these programs into models of service delivery (Harden et al., 2015; Mandell et al., 2013). Further exploration of PF components, such as assessing and monitoring nonspecific program effects (e.g., therapeutic alliance or SSGP-related adverse events) and the participant's enactment of the learned skills, both within and outside of the group context, can clarify whether observed positive effects are attributable to the SSGP or the facilitators running the groups (Bellg et al., 2004; Kang et al., 2021), enabling consideration of how these factors impact on participant attrition and adherence (Borrelli et al., 2005).

### Meta-analysis

As reported by previous reviews (Gates et al., 2017; Wolstencroft et al., 2018), the present meta-analysis revealed a positive effect of SSGPs on social outcomes. However, this finding should be generalised with caution, given these outcomes were largely measured via adolescent self-reported gains in social knowledge (Gates et al., 2017) and included an instrument specifically developed to assess the efficacy of the PEERS® SSGP (Tseng et al., 2020).

Findings from the meta-analysis revealed a large overall effect size comparable to previous studies evaluating the efficacy of SSGPs for autistic youth (Gates et al., 2017; Wolstencroft et al., 2018). Most studies were undertaken with samples where the majority of participants were male, a factor significantly moderating the effects of SSGP (Freitag et al., 2016; Lerner & Mikami, 2012; Rabin et al., 2018; Yoo et al., 2014). Research suggests that the social challenges experienced by autistic males and females differ, and they respond to SSGP differently (Dean et al., 2017), with females possibly benefitting more from attending these programs (Choque Olsson et al., 2017). Considering the higher male-to-female ratio amongst autistic youth (ABS, 2019), there are usually fewer females in SSGP groups. Limited contact with other young females and being in a male-dominated group may limit the social benefits of attending these programs for female autistic youth (Cridland et al., 2014).

### Findings

Studies rarely obtained data from informants reporting on participants’ performance in everyday social contexts such as schools, with those collecting data from teachers failing to find any significant changes following participation in SSGPs (Gates et al., 2017). Future research would benefit from understanding the generalised effects of these interventions in contexts beyond program groups. There is a further limited understanding of the impact of dosage (number of sessions) on program outcomes (Gates et al., 2017).

Wolstencroft et al. (2018) review suggested that well-designed SSGPs improve autistic youths’ social knowledge and performance. Interestingly, findings from the present review identified three further outcome categories commonly utilised in assessing the efficacy of SSGP, including autism characteristics which demonstrated no significant program effect for these programs (as measured by parent proxy reports). This finding contradicts those of previous systematic reviews and meta-analyses reporting a modest efficacy for SSGPs based on this category (Gates et al., 2017; Wolstencroft et al., 2018). The majority of research designs evaluating the efficacy of SSGPs continue to employ measures of autistic characteristics as their primary outcome measure. This approach may be counterproductive, given that it inadvertently promotes the view that being autistic is problematic, compounding participants’ feelings of marginalisation and difference (Gillespie-Lynch et al., 2017). This approach does not align with contemporary views of autism which focus on supporting individual needs rather than promoting the notion that individuals need to comply with the neurotypical world (Cage et al., 2018; Monahan et al., 2021). There is a clear need for future research to focus both on building autistic youth’s social competence and promoting the acceptance of neurodiverse individuals within their social contexts (Bölte et al., 2021).

Adolescence is characterised by periods of emotional instability and growth (Hare et al., 2008), with adolescents commonly spending considerable time by themselves and with their peers and less time with their families (Guivarch et al., 2017). Given that adolescence is a time when young people begin to assert their independence from their parents and that autistic adolescents are the consumers of SSGPs, measuring their perceptions of the social knowledge they gain as a result of attending these programs is important in understanding their true efficacy and impact (Gates et al., 2017). To date, autistic youths’ lived experiences and views on the content and structure of SSGPs are rarely considered (Afsharnejad et al., 2021a, 2021b; Choque Olsson et al., 2016; Lurie & Morgan, 2013; Monahan et al., 2021). Limited understanding of the social skills and norms autistic youth themselves wish to adopt and practice may result in SSGPs inadvertently promoting camouflaging, negatively impacting participants' long-term mental health (Cage et al., 2018; Cassidy et al., 2018, 2020). The efficacy of SSGPs would likely be improved by a greater understanding of the views of autistic youth on neurotypical social norms, the skills they wish to develop, and involving them more actively in co-producing the content and strategies employed in SSGPs (Björling et al., 2020; Monahan et al., 2021).

### Other Findings

As found by previous reviews, the majority of SSGPs were delivered weekly by health professionals in sessions over 90 min to small groups of 3 to 10 autistic youth. The number of sessions within each program predominantly ranged from 12 to 16 sessions. Notably, SSGPs aiming to support the social skills of autistic adolescents vary in regard to their aims and approaches, which range from structured, semi-structured, and unstructured to performance-based. This variability may, in part, be in response to the preferences and goals of autistic youth and the target context and culture. Consideration of the appropriateness of SSGP for autistic youth should include an assessment of the alignment between a particular program and the target setting and culture (Marsiglia & Booth, 2015). To date, few SSGPs have incorporated adolescents' personally meaningful social goals or in-vivo activities outside their sessions' regular settings. It is plausible that providing opportunities for youth to practice their skills in everyday social situations would increase the effects associated with SSGPs (Afsharnejad et al., 2021a, 2021b).

Across studies, the limited reporting of autistic adolescents’ level of motivation for engaging in SSGPs prior to their enrolment is cause for concern, particularly given the evidence that participants' motivation strongly influences the outcomes of these programs (Chevallier et al., 2012). Despite some programs employing goal setting as a strategy to enhance the intrinsic motivation of participants to engage in SSGPs, only one study reported actively involving participants in establishing their goals. Failing to support the autonomy of autistic adolescents in identifying and pursuing their own social goals can negatively impact the outcomes of SSGPs (Hodgetts et al., 2018).

Across this body of evidence, there is limited understanding of the impact of dosage (number of sessions) on program outcomes (Gates et al., 2017). To date, only efficacy evaluations of KONTAKT® have considered the impact of dose on the outcomes of autistic youth, finding that a longer, 24-session variant demonstrated nearly twice the effect of a medium 16-session and shorter, 12-session variants (Afsharnejad et al., 2021a, 2021b; Choque Olsson et al., 2017; Jonsson et al., 2018). Although this research provides preliminary insight into the role of dosage in influencing outcomes, there is a need to further understand the efficacy of SSGPs across other cultural and service delivery contexts and the feasibility of these longer programs, given their cost (Wolstencroft et al., 2018). Given these limitations and the continued development of novel SSGPs, the external validity of the programs would be improved by efficacy evaluations with more heterogeneous (Gates et al., 2017) and community-sourced samples.

Lastly, though this review enabled insights into the design of studies published over 12 years evaluating the efficacy of SSGPs via an RCT design, it must be noted that autism-related research is rapidly evolving. As such, the SSGPs evaluated, study methods and approaches do not reflect some of the more contemporary stances in this field, such as the neurodiversity paradigm (Lord et al., 2021). More recent research has suggested that social interaction difficulties experienced by autistic individuals may be due to a mismatch in communication styles between autistic and non-autistic neurotypes (Fletcher-Watson et al., 2019). As such, questions may arise about the appropriateness of SSGPs for autistic individuals. Especially given the potential for unattended and adverse effects (i.e., as a result of masking). Nevertheless, SSGPs are still frequently implemented in clinical settings. These programs likely remain helpful for some individuals to develop the skills they need to meet their social goals and communicate their wants and needs. By nature, a review can only examine what has been done in the past. Going forward, researchers should focus on developing SSGPs co-produced with autistic individuals to enhance the relevance and efficacy of such programs. Likewise, programs that support neurotypicals better communicate with other neurotypes may also be important.

### Limitations

This systematic review has several limitations. The meta-analysis results represent 72% of the 18 studies included in this systematic review, posing a risk for potential publication bias. Further, nearly half of the identified studies failed to report the total scores of outcome measures, reporting only subscale scores, with only half of the relevant body of research contributing to the meta-analysis. Variation across the measurement frameworks employed by studies (Gates et al., 2017; Wolstencroft et al., 2018) necessitated categorising outcome measures into four key areas, likely limiting the conceptual clarity of the meta-analysis and contributing to the high heterogeneity. Despite the necessity for assessing and reporting publication bias, the meta-analysis of the outcome categories and the maintenance effect, which contain less than 10 studies, are less reliable due to low statistical power (Dalton et al., 2016). The practice of pooling outcomes across autistic children and adolescents (Choque Olsson et al., 2017; Corbett et al., 2016, 2019; Freitag et al., 2016; Jonsson et al., 2018) combined with findings suggesting adolescents’ experience greater gains from attending SSGPs than children (Choque Olsson et al., 2017) raises the question that this meta-analysis does not solely reflect the efficacy of SSGPs in autistic adolescents alone. There was variability across the included studies with regard to the delivery of SSGPs, program components, participants, and data collection approaches, which is not unusual in the context of a systematic review and meta-analysis. Studies aligned in terms of their aims and fundamental principles, with the majority evaluating standardised SSGPs (Tseng et al., 2020).

The delivery of SSGPs is affected by the training and skills of program facilitators (Craig et al., 2008). Further, allocation concealment for studies evaluating the efficacy of behavioural interventions such as SSGPs can be difficult, if not impossible. Given the noted limitations in the quality of the design of RCTs reviewed, the robustness of future efficacy evaluations of the programs would be significantly improved by adhering to guidelines such as CONSORT (Boutron et al., 2008). Due to the lack of response from corresponding authors, the PF was scored based on the information provided in the published RCT manuscripts. Although it is plausible that the limited word counts allowed by publishers contributed to items being marked as “not reported” on the PF checklist, future studies should strongly consider reporting PF items.

In assessing PF, previous studies have largely relied on assessment methods with low reliability (e.g., observational methods or checklists; Borrelli, 2011). Future research should consider employing more rigorous approaches to fidelity assessment, including capturing the views of all stakeholders in relation to a SSGP and audio/video recording sessions, enabling evaluation of the reliability and validity of reported adherence (Borrelli, 2011). Social skills are complex, a fact that has likely underpinned the evidential lack of consistency in the measurement frameworks employed across SSGP efficacy studies. In the present review, variability across studies concerning their choice of outcome measurements did not allow assessment of how PF influences the efficacy of a SSGP.

Few studies employed active control groups in their designs, enabling control for exposure to social context. Although the vast majority of evidence assessing the efficacy of SSGPs for autistic adolescents is underpinned by comparison to inactive control groups, the effects of SSGPs relative to other social groups remain unclear (Karlsson & Bergmark, 2015). As RCT studies are associated with high levels of missing data and noncompliance, future evaluations employing intention-to-treat approaches to data analysis can maintain the balance across study arms achieved by randomisation (Gupta, 2011) regardless of participant withdrawals (Everitt & Wessely, 2008). Given the contextual and highly individualised nature of social skills in autistic youth (Marsiglia & Booth, 2015), future research should consider employing single-subject research designs to advance understanding of the efficacy of SSGPs at the individual rather than group level.

## Conclusions

This review found that despite demonstrating good to strong methodological quality, the majority of studies assessing the efficacy of SSGPs neglect clear reporting of PF, negatively impacting both their internal validity (i.e., the extent to which the implementation of the program aligned with study protocols) and external validity (i.e., the extent to which a study can be replicated and interpreted in a real-world context). Although previous reviews have concluded that SSGPs have a significantly moderate effect on the outcomes of autistic youth, the present review, which categorised outcomes into four discrete groups, is the first to highlight that these effects can be largely attributed to changes in social functioning with SSGPs having limited effect on autistic traits and behavioural and emotional challenges. The findings of this review highlight the need for existing SSGPs targeting autistic youth to consider factors affecting PF, accounting for the effect of a supportive group context and program dosage, and considering the alignment of these programs with the social goals of autistic adolescents themselves. Further investigations evaluating the efficacy of carefully conceptualised and designed SSGPs should attempt to accommodate for the heterogeneity of the autism spectrum and variations in a social context while attending to methodological fidelity.

## Appendix 1

See Tables 4, 5 and 6.

**Table 4:** Description of programs targeting social competence evaluated via a randomised controlled trial

Program	Description	Aim	Principles	Components	Targeted skills	Session format
KONTAKT®^a^	Structured manualised SSGP	Improve social interaction and motivation, communication, awareness of self and others, problem-solving, and self-confidence	CBT, computer-based cognitive training, behavioural activation, psychoeducation, observational learning, and parent involvement	Individualised goals, group rules, group discussions, role play, emotion-processing training, group activities, tailored homework, and unstructured socialisation. Extended version: leading a session (including activities and discussion), activities outside the clinic (real-life practice)	Verbal and nonverbal Conversational skills, learning about autism, group situations, understanding oneself and others, handling misunderstandings, disagreements, sarcasm, rejection, teasing & bullying, humour, new situations, staying in contact	Opening the session, Homework assignment follow-up, group exercise, snack time, Group discussion, homework assignment, closing the session
MASSI^b^	Structured psychosocial program	Promoting social skills development	CBT, psychoeducation, ABA, Individual & group therapy, parent education, didactic teaching	Individualised plan, emphasis on corrective, positive social learning experiences, modelling new skills, feedback, and explicit teaching about autism and anxiety	Verbal and nonverbal Conversational skills, learning about autism and anxiety, group situations, emotion regulation, handling rejection	NA
PEERS®^c^	Manualised parent-assisted SSGP	Learning how to make and maintain friends	Psychoeducation, CBT, didactic teaching, parent involvement (PwP: peer mediation)	Homework assignments, concrete behavioural rules, modelling and role-play, practising the newly learned skill, individualised homework	Conversational and friendship skills, handling misunderstandings, disagreements, sarcasm, rejection, teasing, bullying, bad reputations, rumours & gossip, humour, good sportsmanship, get-togethers	Review homework (P/T), parent education (P), social skills lesson (role-play & feedback) (P), Assigning new Homework (P), review the session (P & T)
SDARI^d^	Unstructured performance-based SSGP	Experience in successful engagement in social skills. motivating interactions	Social performance approach, with minimal didactic teaching	Structured and unstructured activities	Verbal and nonverbal conversational skills, understanding oneself and others, Cooperation, self-regulation	Activities, free play break, games
SENSE Theatre®^e^	A semistructured theatre-based program	Improve interaction with peers in natural settings and take part in two public performances	Experiential techniques, peer mediation, behavioural techniques	Modelling, directed verbal and nonverbal communication, role-play, improvising, play performance, imaginative play, and interacting with trained NT youth. Roles assigned by program directors (based on age, verbal ability, participant interests, and special talents)	NA	NA
SOSTA-FRA^f^	Highly structured manualised SSGP	Improve social motivation, social cognition, self-regulation, and social interaction with peers	CBT, operant behavioural techniques, computer-based training, observational techniques, parent involvement	Group rules, session-specific individual goals, guided discussions, focussing on individual experience, role-play with feedback), group play, computer-based emotion recognition, training of daily situations, homework	Verbal and nonverbal conversational skills, group situations, understanding oneself and others, handling teasing & bullying, social problem solving, goal setting, self & emotion regulation	opening group round, homework discussion, session-specific topic discussion, games, session-specific homework, Closing round with feedback
START^g^	Socialisation program	Improve social knowledge, motivation, and social skills	Experiential and didactic techniques, peer mediation	Discussions, social goals, unstructured socialisation, role play, structured activities, social homework, individually targeted social skills	Verbal and nonverbal conversational skills, group situations, friendship skills, handling misunderstandings, disagreements, sarcasm & rejections, humour, coping strategies, empathy, good sportsmanship, cooperation, phone, and social media, get-togethers	Individual therapeutic check-in session, a group unstructured socialisation time, a group discussion and practice of a social skill topic, a structured group activity, and an individual checkout session with parent involvement

*CBT* cognitive behavioural therapy, *PwP* PEERS® with Peers, *ABA* applied behaviour analysis

^a^Bölte (2018)

^b^White et al. (2010)

^c^Laugeson and Frankel (2010)

^d^Lerner and Levine (2007)

^e^Corbett et al. (2011)

^f^Cholemkery and Freitag (2014)

^g^Vernon et al. (2016)

**Table 5:** Quality, design, eligibility criteria, setting, dosage and duration, stakeholders and costs of studies (k = 18) evaluating the efficacy of a social skills group training program for autistic youth via an RCT design

First author (year)/country	Design	Quality	Eligibility	Setting characteristics	Dosage, duration	Stakeholders, n per group (characteristic)	Cost of attendance/incentives
Afsharnejad et al. (2021a, 2021b) Australia	Two armed parallel RCT SS calculated based on previous studies of the program (*N* = 90) Exp: KONTAKT® (*n* = 46) Con: Active control cooking group (*n* = 44)	MQ^a^ = 96% PF^b^ = 0.92 CONSORT diagram available-	Inclusion: Age: 12–17 years old (M/F = 65/25) IQ > 70 (WASI-II) Diagnosis: Autism, AS, Atypical autism, PDD-NOS (based on DSM-IV/5) confirmed by ADOS Exclusion: Insufficient English language skills, low expressed intrinsic motivation to attend, evidence of rule-breaking and/or aggressive behaviours (confirmed by CBCL), a self-reported history of severe clinically assessed self-injury, conduct disorder, antisocial personality disorder, borderline personality disorder, or any form of schizophrenia or related psychotic disorder	4 centres from the Autism Association of Western Australia	16 weekly sessions 90 min (AA) 3 × 90-min concurrent PAA sessions	4–8 (AA) 19 (trained and supervised Occupational therapist [*n* = 12], psychologist [*n* = 2], educators [*n* = 3], social workers [*n* = 1], and speech therapists [*n* = 1])	Free/No
Choque Olsson et al. (2017) Sweden	Two armed parallel RCT SS calculated based on a pilot study (*N* = 296) Exp: KONTAKT® (*n* = 150) Con: Usual care (*n* = 146)	MQ^a^ = 88% PF^b^ = 0.76 CONSORT diagram available	Inclusion: Age: 7–17 years old (M/F = 208/88) IQ > 70 (WISC-IV) Diagnosis: Autism, AS, Atypical autism, PDD-NOS (based on ICD-10) confirmed by ADOS Motivation to participate Exclusion: Clinically assessed self-injury, conduct disorder, anti-social personality disorder, borderline personality disorder, and any psychotic disorder, Insufficient Swedish language skills	12 Outpatient units and one academic clinical outpatient unit	12 weekly sessions 90 min (AA) /60 min (AC) 3 × 90-min concurrent PAA sessions	4–8 (AA/AC) 2 (trained and supervised psychologists [*n* = 39], social workers [*n* = 5], nurses [*n* = 5], special educators [*n* = 2] and speech therapists [*n* = 1])	NR /$12 Voucher
Corbett et al. (2016) USA	Two armed parallel RCT SS not calculated (*N* = 30) Exp: SENSE Theatre® (*n* = 14) Con: Waitlist (*n* = 16)	MQ^a^ = 81% PF^b^ = 0.54 CONSORT diagram available	Inclusion: Age: 8–14 years old (M/F = 24/6) IQ > 70 (WASI) Diagnosis: ASD (based on DSM-5) confirmed by ADOS	University School of Nashville	10 weekly sessions 240 min	NR (AA/AC) NR (trained and supervised staff [*n* = NR]) 12 (trained and supervised NTA, *M*_age_ = 15.33 years old, *SD* = 1.12, [*n* = NR])	Free/NR
Corbett et al. (2019) USA	Two armed parallel RCT SS not calculated (*N* = 77) Exp: SENSE Theatre® (*n* = 44) Con: Waitlist (*n* = 33)	MQ^a^ = 81% PF^b^ = 0.62 No CONSORT diagram	Inclusion: Age: 8–16 years old (M/F = 59/18) IQ > 70 (WASI) Diagnosis: ASD (based on DSM-5) confirmed by ADOS Exclusion: Aggressive behaviours (verbal or physical threats of harming other	A School auditorium	10 weekly sessions 240 min	NR (AA/AC) NR (trained and supervised staff [*n* = NR]) 12 (trained and supervised USN Theatre Guild members [*n* = NR])	Free/NR
Freitag et al. (2016) Germany	Two armed parallel RCT SS calculated based on a pilot study (*N* = 209) Exp: SOSTA-FRA (*n* = 107) Con: Usual care (*n* = 102)	MQ^a^ = 81% PF^b^ = 0.51 No CONSORT diagram	Inclusion: Age: 8–19 years old (M/F = 186/15) IQ > 70 (WISC/WAIS) Diagnosis: Autism, AS, Atypical autism (based on ICD-10) confirmed by ADOS/ADI-R Fluency in German Exclusion: Schizophrenia, bipolar disorder, social phobia, OCD, MDD with suicidal thoughts, psychiatric disorders, aggressive behaviour, neurological disorders (except well-treated epilepsy), medical conditions interfering with therapy, exposure to SSGPs (< 6 months)	Six German University Departments and an outpatient clinic	12 weekly sessions 90 min (AA) /60 min (AC) 3 × 90-min concurrent PAA session	4–5 (AA/AC) 2 (CBT-trained therapists [*n* = NR], supervision NR)	NR/NR
Jonsson et al. (2018) Sweden	Two armed parallel RCT SS not calculated (*N* = 50) Exp: KONTAKT® (*n* = 23) Con: Usual care (*n* = 27)	MQ^a^ = 96% PF^b^ = 0.82 CONSORT diagram available	Inclusion: Age: 7–17 years old (M/F = 35/15) IQ > 70 (WISC-III/IV) Diagnosis: ASD (based on ICD-10) confirmed by ADOS Motivation to participate Exclusion: Clinically assessed self-injury, conduct disorder, anti-social borderline personality disorder, psychotic disorders, Insufficient Swedish language skills	psychiatry outpatient unit and an academic clinical outpatient unit	24 weekly sessions 90 min (AA) /60 min (AC) 3 × 90-min concurrent parent session	4–8 (AA/AC) 2–3 (trained and supervised Psychologists [*n* = 7])	NR/€11 Voucher
Ko et al. (2019) USA	Two armed parallel RCT SS not calculated (*N* = 40) Exp: PEERS® (*n* = 20) Con: Waitlist (*n* = 20)	MQ^a^ = 96% PF^b^ = 0.56 No CONSORT diagram	Inclusion: Age: 12–17 years old (M/F = 24/11) IQ > 70 (KBIT) Diagnosis: ASD (based on DSM-5) diagnosis not confirmed Communicate/comprehend full sentences; mild/severe autism characteristics	NR	20 weekly sessions 90 min (AA) 5–10 min (PAA)	4–6 (AA) NR (trained and supervised undergraduate research assistants [*n* = 4]) NR (trained and supervised NTA volunteers)	NR/NR
Laugeson et al. (2009) USA	Two armed parallel RCT SS not calculated (*N* = 33) Exp: PEERS® (*n* = 17) Con: Waitlist (*n* = 16)	MQ^a^ = 77% PF^b^ = 0.43 No CONSORT diagram	Inclusion: Age: 13–17 years old (M/F = 28/5) IQ > 70 (K-BIT) Diagnosis: High functioning autism, AS, PDD-NOS (based on ICD-10) diagnosis not confirmed Fluency in English No history of major mental illness (e.g., bipolar disorder, schizophrenia, or psychosis), hearing, visual, or physical impairments	NR	12 weekly sessions 90 min (AA/PAA)	7 (AA) NR (trained and supervised psychologists [*n* = NR]) 1 (trained and supervised coaches, Psychology postgraduate student Research assistant [*n* = NR])	NR/Paid parking, brief evaluation summary, weekly light meal with beverages
Lerner et al. (2012) USA	Two armed parallel RCT SS not calculated (*N* = 13) Exp: SDARI (*n* = 7) Con: Active control (Skill streaming; *n* = 6)	MQ^a^ = 77% PF^b^ = 0.53 No CONSORT diagram	Inclusion: Age: NR (M/F = 13/0) IQ: NR Diagnosis: Autism, AS, PDD-NOS (based on ICD-10) confirmed by SCQ & SRS	NR	4 weekly sessions 90 min	7 (AA) NR (trained and supervised staff [*n* = 3])	NR/NR
Matthews et al. (2018) USA	Three armed parallel RCT SS not calculated (*N* = 34) Exp: PEERS® with peers (*n* = 12) Con 1: Active control (traditional PEERS®, *n* = 10) Con 2: Waitlist (*n* = 12)	MQ^a^ = 85% PF^b^ = 0.52 No CONSORT diagram	Inclusion: Age: 13–17 years old (M/F = 28/6) Verbal IQ > 70 (K-BIT) Diagnosis: Autism, ASD (based on DSM-IV/5) confirmed by ADOS > 80% education in the general education setting, difficulty making friends according to parent report, parent willingness to be involved Exclusion: Home-schooled or online students	A community-based non-profit autism centre	14 weekly sessions 90 min (AA/PAA)	NR (AA) 3 (trained and supervised postgraduate counsellors [*n* = 3] and undergraduate clinicians [*n* = 1]) NR (trained and supervised NTA peers with no diagnosed disorders [*n* = 8])	NR/$150 Voucher
Matthews et al. (2020) USA	Two armed parallel RCT SS not calculated (*N* = 21) Exp: Accelerated PEERS® (*n* = 11) Con: Active control (traditional PEERS®; *n* = 10)	MQ^a^ = 77% PF^b^ = 0.31 No CONSORT diagram	Inclusion: Age: 13–17 years old (M/F = 17/4) Verbal IQ > 70 (K-BIT) Diagnosis: ASD (based on DSM-IV /5) confirmed by ADOS > 80% in the general education setting, difficulty making friends according to parent report, parent willingness to be involved	A community-based non-profit autism centre	14 weekly sessions 90 min (AA/PAA)	5 (AA) 3 (trained and supervised Master’s and Bachelor’s level practitioners [*n* = NR]) NR (trained and supervised NTA [*n* = NR])	NR/NR
Rabin et al. (2018) Israel	Two armed parallel RCT SS not calculated (*N* = 41) Exp: PEERS® (*n* = 20) Con: Waitlist (*n* = 21)	MQ^a^ = 88% PF^b^ = 0.30 No CONSORT diagram	Inclusion: Age: 12–17 years old (M/F = 39/2) IQ > 70 (WISC-III/IV) Diagnosis: ASD (based on ICD-10) confirmed by ADOS Motivation to participate Born in Israel & Speak Hebrew No severe behavioural problems; Parents willingness to be a social coach and attend all sessions	NR	16 weekly sessions 90 min (AA/PAA)	NR	NR/Voucher for Parents and teachers (amount NR)
Schohl et al. (2014) USA	Two armed parallel RCT SS not calculated (*N* = 58) Exp: PEERS® (*n* = 25) Con: Waitlist (*n* = 33)	MQ^a^ = 81% PF^b^ = 0.36 CONSORT diagram available	Inclusion: Age: 11–16 years old (M/F = 47/11) Verbal IQ > 70 (K-BIT) Diagnosis: ASD, confirmed by ADOS Motivation to participate Fluency in English No history of major mental illness (e.g., bipolar disorder, schizophrenia, or psychosis), hearing, visual, or physical impairments	NR	14 weekly sessions 90 min (AA/PAA)	≤ 10 (AA) 3 (trained and supervised postgraduate psychology students [*n* = 5]) 1 (trained and supervised undergraduate research assistants [*n* = NR])	Free/$30 Voucher
Shum et al. (2019) China	Two armed parallel RCT SS not calculated (*N* = 72) Exp: PEERS® (*n* = 43) Con: Waitlist (*n* = 29)	MQ^a^ = 85% PF^b^ = 0.35 CONSORT diagram available	Inclusion: Age: 11–15 years old (M/F = 57/15) Verbal IQ > 70 (WISC-IV) Diagnosis: Autistic disorder, AS, ASD, PDD-NOS (based on ICD-10) confirmed by ADOS Fluency in Cantonese Studying at a high school in Hong Kong Exclusion: Low motivation, ADOS < 5, other medical problems, previous exposure to PEERS®	At community service centres	14 weekly sessions 90 min (AA/PAA)	≤ 10 (AA) 4 (trained and supervised social workers, speech therapists or occupational therapists [*n* = 18]) NR (trained and supervised Psychology undergraduate student coaches [*n* = NR])	NR/NR
Van Hecke et al. (2015) USA	Two armed parallel RCT with case-control SS not calculated (*N* = 79) Exp: PEERS® (AA, *n* = 35) Con: Waitlist (AA, *n* = 31) Case-control (NTA, *n* = 30)	MQ^a^ = 81% PF^b^ = 0.44 CONSORT diagram available	Inclusion (AA/NTA): Age: 11–16 years old (M/F = 67/12) Verbal IQ > 70 (WISC-IV) Motivation to make friends Fluency in English No neural, physical, hearing, or visual impairments, bipolar disorder, or schizophrenia co-occurrence; and enrolment in middle/high school or having weekly peers contacts outside of the family, if home-schooled Additional requirements for AA: Diagnosis: Autism, ASD (based on the DSM-IV) confirmed by ADOS Attending at least 12 sessions Additional requirements for NTA: ASSQ < 13 and CBCL < 65	NR	14 weekly sessions 90 min (AA/PAA)	NR (AA) NR (trained and supervised postgraduate psychology students [*n* = NR]) NR (trained and supervised Psychology undergraduate student coaches [*n* = NR])	Free/$30 Voucher
Vernon et al. (2018) USA	Two armed parallel RCT SS not calculated (*N* = 40) Exp: START (*n* = 21) Con: Waitlist (*n* = 19)	MQ^a^ = 85% PF^b^ = 0.44 CONSORT diagram available	Inclusion: Age: 12–17 years old (M/F = 24/11) Verbal IQ > 70 (K-BIT) Diagnosis: Autism, AS, Atypical autism, PDD-NOS, confirmed by ADI-R Exclusion: Not having an ASD diagnosis	NR	20 weekly sessions 90 min (AA) 5–10 min (PAA)	3–6 (AA) 2–4 (trained and supervised undergraduate research assistants [*n* = NR]) 1–2 (trained and supervised NTA volunteers [*n* = NR])	NR/NR
White et al. (2013) USA	Two armed parallel RCT SS not calculated (*N* = 30) Exp: MASSI (*n* = 15) Con: Waitlist (*n* = 15)	MQ^a^ = 88% PF^b^ = 0.44 CONSORT diagram available	Inclusion Age: 12–17 years old (M/F = 23/7) Verbal IQ > 70 (measure NR) Diagnosis: Autism, AS, PDD-NOS, confirmed by ADOS Exclusion: Primary diagnosis of OCD, special phobias, panic disorder with/without Agoraphobia, Serious behaviour issues	NR	13 (In)/7 (G) weekly sessions 60–75 min	3 (AA) 1 (trained and supervised psychologists [*n* = 1] and postgraduate psychology students) NR (trained and supervised NTA volunteers [*n* = NR])	NR/NR
Yoo et al. (2014) Korea	Two armed parallel RCT SS not calculated (*N* = 47) Exp: PEERS® (*n* = 23) Con: Waitlist (*n* = 24)	MQ^a^ = 88% PF^b^ = 0.47 No CONSORT diagram	Inclusion: Age: 12–18 years old (M/F = 44/3) Verbal IQ > 65 (WISC-IV) Diagnosis: Autistic disorder, AS, PDD-NOS, confirmed by ADOS Motivation to participate Fluency in Korean No history of major mental illness, current aggressive behaviour or severe oppositional tendency, and no hearing, visual, physical, or neurological disabilities	NR	14 weekly sessions 90 min (AA/PAA)	6–10 (AA) NR (trained and supervised Psychiatrists, psychologists, special educators [*n* = 6]) NR (trained and supervised coaches [*n* = 3])	NR/NR

*AA* autistic adolescent, *AC* autistic children, *ADI-R* Autism Diagnostic Interview-Revised, *ADOS* Autism Diagnostic Observation Schedule, *AS* Asperger syndrome, *ASD* autism spectrum disorder, *ASSQ* Autism Spectrum Screening Questionnaire, *CBCL* Child Behaviours Checklist, *CBT* cognitive behavioural therapy, Con control group, *DSM-IV* Diagnostic and Statistical Manual of Mental Disorders—Fourth Edition, *DSM-5* Diagnostic and Statistical Manual of Mental Disorders-Fifth Edition, *Exp* experiment group, *F* female, *G* group sessions, *ICD-10* International Statistical Classification of Diseases and Related Health Problems-10th Edition, *PF* program fidelity, In Individual sessions, *K-BIT* Kaufman Brief Intelligence Scale, *M* male, *MASSI* Multimodal Anxiety and Social Skill Program, *MDD* major depression disorder, *MQ* methodological quality, *NR* not reported, *NTA* neurotypical adolescents, *OCD* obsessive–compulsive disorder, *PAA* parents of autistic adolescents, *PDD-NOS* pervasive developmental disorder, *PEERS*® Program for the Education and Enrichment of Relationship Skills, *SCQ* Social Communication Questionnaire, *SDARI* Sociodramatic Affective Relational Program, *SS* sample size, *SSGP* social skills group program, *START* Social Tools and Rules for Teens, *USN* University School of Nashville, *WASI* Wechsler Abbreviated Scale of Intelligence, *WAIS* Wechsler Adult Intelligence Scale, *WISC-III* Wechsler Intelligence Scale for Children-Third Edition, *WISC-IV* Wechsler Intelligence Scale for Children-Forth Edition

^a^Assessed via Kmet Checklist (Kmet et al., 2004) with scores of > 80% demonstrating strong, 70–80% good, 50–70% adequate and < 50% limited methodological quality (Lee et al., 2008)

^b^Assessed via Treatment Fidelity Assessment and Implementation Plan with higher scores demonstrating higher adherence to the study’s protocol (Borrelli, 2011)

**Table 6:** The outcomes and findings of studies (k = 18) evaluating the efficacy of a social skills group training program for autistic youth via an RCT design

References	Assessment Timepoints	Outcome measures	Other measures	Attrition rate (%)	Data analysis	Results post SSGP
Afsharnejad (2017)	Baseline, post-program and 12-week follow-up	AA: GAS^b, p^, CSIE, PALs, SIAS, PedsQL, ESM PAA: ERSSQ, PedsQL, SRS, ESM R: GAS^b^, Emotion recognition task^b^	Adverse events, feedback, semistructured interviews Fidelity: Checklist & coding video recordings	11.1	Intent-to-treat approach Linear mixed effect	Achievement of personally meaningful social goals; Decreased social interaction anxiety at follow-up (primary endpoint)
Choque Olsson et al. (2017)	Baseline, post-program and 3-month follow-up	AA: CiS PAA: ABAS-II, PSS, SRS^p^ R: CGI-S, DD-CGAS T: ABAS-II, SRS^b, p^	Adverse event Fidelity: Checklist & coding video recordings	15	Intent-to-treat approach Linear mixed effect	Decreased autism characteristics for adolescents only, especially in females
Corbett et al. (2016)	Baseline, post-program and 2-month follow-up (Some measures)	PAA: ABAS-II, SRS R: ERP, NEPSY-II (MFI, MFD^p^, ToM), PIP^b^	Fidelity: Observation & rating scales	9.1	ANCOVA	Improved social cognition, memory for faces and group play: Decreased autism characteristics
Corbett et al. (2019)	Baseline, post-program	R: ERP, NEPSY-II (ToM), PIP	Fidelity Scale	11.5	ANCOVA	Improved verbal ToM and engagement in cooperative play
Freitag et al. (2016)	Baseline, post-program and 3-month follow-up	AA: DIKJ, Therapy quality & achievements PAA: CBCL, SDQ, SRS^p^ T: SDQ, SRS^b^	Adverse event collected Fidelity: Coding video recordings	7.5	Intent-to-treat approach Mixed Model Repeated Measures	Decreased autism characteristics Results maintained to follow-up
Jonsson et al. (2018)	Baseline, post-program and 12-week follow-up	PAA: ABAS-II, PSS, SRS^p^ R: CGI-S, DD-CGAS T: ABAS-II, SRS^b, p^	Adverse event collected Fidelity: Checklist & coding video recordings	16	Intent-to-treat approach Linear mixed effect	Decreased autism characteristics; Improved global functioning SRS results maintained to follow-up
Ko et al. (2019)	Baseline, post-program	R: Observation^p^ (facial expression, social interaction, social inquiries^b^)	Fidelity: Coding a checklist during the session	12.5	ANOVAs	Improved social interaction (number of questions asked; positive facial expressions)
Laugeson et al. (2009)	Baseline, post-program	AA: FQS, QPQ, TASSK-R PAA: QPQ, SSRS T: SSRS^b^	Fidelity: Coding a checklist during the session	0	Mixed MANOVA	Improved social skills, friendship knowledge, and friendship quality; Increased hosted get-togethers
Lerner et al. (2012)	Baseline, post-program	PAA: SRS^b, p^, SSRS^b, p^ R: SIOS^p^, sociometric nomination^p^ T: SSRS^p^	Fidelity: Coding a checklist during the session	0	Repeated-measures ANOVA	Both experiment and active control increased reciprocated friendship and social skills, with the latter not generalised to daily life
Matthews et al. (2018)	Baseline, post-program and 20-week follow-up	AA: AKQ, QSQ, R-UCLA, SIAS, TASSK PAA: QSQ, SRS, SSIS	Fidelity: Coding a checklist during the session	15.9	MANOVA models	Increased social skills’ knowledge and social functioning compared to WLC; AC hosted more get-togethers compared to WLC; No difference between the EXP & Con 1 Results maintained to follow-up
Matthews et al. (2020)	Baseline, post-program	AA: QSQ, TASSK PAA: QSQ, SRS, SSIS	Fidelity assessed	NA	Wilcoxon signed-rank	Decreased autism characteristics; Increased social skills knowledge
Rabin et al. (2018)	Baseline, post-program and 4-month follow-up	AA: EQ, LSDQ, QSQ, TASSK PAA: QSQ, SRS, SSIS R: CASS^b^ T: SRS, SSIS	Fidelity: NR	2.5	Repeated measure MANOVA	Improvements in engagement, question-asking, physical arousal, social skills, social encounters, empathy, and social-skill knowledge; Decreased autism characteristics Results maintained to follow-up
Schohl et al. (2014)	Baseline, post-program	AA: FQS, QSQ, TASSK, SIAS PAA: QSQ, SRS, SSRS T: SRS^b^, SSRS^b^	Fidelity: Coding a checklist during the session	7.9	ANCOVA	Improved friendship skills and knowledge
Shum et al. (2019)	Baseline, post-program and 14-week follow-up	AA: QPQ, TASSK PAA: ABAS, QPQ, SRS T: SRS^b^ NTA: ASBS^b^	Fidelity: Observing the session	8.3	Repeated measure MANOVA	Improved social skills’ knowledge, and social communication; Decreased autism characteristics Results maintained to follow-up
Van Hecke et al. (2015)	Baseline, post-program	AA: TASSK PAA: QSQ, SRS R: EEG	Fidelity: Coding a checklist during the session	9.4	Repeated measures ANOVA	A shift from right to left hemisphere associated with more social contacts and knowledge; Decreased autism characteristics
Vernon et al. (2018)	Baseline, post-program	AA: SMCS, SSIS PAA: SMCS, SSIS, SRS	Fidelity: Coding a checklist during the session	12.5	Mixed MANOVA	Increased social competencies and global social functioning; Reduced autism-related social vulnerabilities and autism characteristics
White et al. (2013)	Baseline, post-program	AA: Program satisfaction PAA: CASI-Anx^p^, program satisfaction rating, SRS^p^ R: CGI-I^b^, DD-CGAS^b^, PARS^b^	Adverse event: Only collected if reported by the participants Fidelity: Checklist & coding video recordings	16.7	Intent-to-treat approach ANCOVA	Decreased autism characteristics and anxiety level
Yoo et al. (2014)	Baseline, post-program and 3-month follow-up (some measures)	AA: TASSK^p^, QPQ^p^, SSRS, SCQ^p^, CDI, STAIC PAA: BDI, STAIC, ASDS, QPQ, SRS^p^, ASDS, CBCL, EHWA-VABS ^p^ R: ADOS^p^	Fidelity: Observing the session	14.5	Repeated measures ANOVA	Improved interpersonal relationship and play/leisure time, social skills knowledge; Decreased depressive symptom Results maintained to follow-up

*AA* autistic adolescents, *ABAS-II* adaptive behaviour assessment system II, *ADOS* autism diagnostic observation schedule, *AKQ* autism knowledge questionnaire, *ANCOVA* analysis of covariance, *ANOVA* analysis of variance, *ASBS* adolescent social behaviour scale, *ASDS* Asperger syndrome diagnostic scale, *BDI* beck depression inventory, *CASI-Anx* child and adolescent symptom inventory-4 anxiety scale, *CASS* contextual assessment of social skills, *CBCL* child behaviour checklist, *CDI* child depression inventory, *CGI-S* Ohio State University Autism Clinical Global Impression-Severity, *CiS* children in stress, *CSIE* circumplex scale of interpersonal efficacy, *DD-CGAS* developmental disabilities children’s global assessment, *DIKJ* Depressioninventar fur Kinder und Jugendliche, *EHWA-VABS* Korean version of the Vineland Adaptive Behaviour Scale, *EQ* emotion quotient, *ERP* event-related potential, *ESM* experience sampling method, *GAS* goal attainment scaling, *FQS* friendship qualities scale, *LSDQ* loneliness and social dissatisfaction questionnaire, *MANOVA* multivariate analysis of variance, *MFI* memory for faces (Immediate), *MFD* memory for faces (Delayed), *NR* not reported, *PAA* parents of the autistic adolescents, *PALs* Perth A-Loneness scale, *PARS* pediatric anxiety rating scale, *PedsQL* paediatric quality of life inventory TM, version 4.0, *PIP* peer interaction paradigm, *PSS* perceived stress scale, *QPQ* quality of play questionnaire, *QSQ* quality of socialization questionnaire, *R* research team, *R-UCLA* Revised UCLA Loneliness Scale, *SCQ* social communication questionnaire, *SDQ* strength and difficulties questionnaire, *SIAS* social interaction anxiety scale, *SIOS* social interaction observation system, *SMCS* social motivation and competencies scale, *SRS* social responsiveness scale, *SSGP* social skills group program, *SSIS* social skills improvement scale, *SSRS* social skills rating system, *STAIC* the trait anxiety inventory, *T* teachers, *TASSK-R* test of adolescent social skills knowledge-revised, *ToM* theory of mind

^b^Blind assessment

^p^Primary outcome measure

## Footnotes

1 To describe individuals diagnosed with autism spectrum disorder, throughout this manuscript, autistic adults’ preferred terminology “autistic” has been used (Kenny et al., 2016).

## References

[CR1] Achenbach TM, Edelbrock CS (1978). The classification of child psychopathology: A review and analysis of empirical efforts. Psychological Bulletin.

[CR2] *Afsharnejad B., Falkmer M., Black M.H., Alach T., Fridell A., Coco C., Milne K., Bölte, S. & Girdler, S. (2021a). KONTAKT® social skills group training supports autistic adolescents in achieving their personally meaningful social goals: A randomised actively controlled trial. *European Child & Adolescent Psychiatry.*10.1007/s00787-021-01814-6

[CR3] Afsharnejad B, Falkmer M, Picen T, Black MH, Alach T, Fridell A, Coco C, Milne K, Bölte S, Girdler S (2021). “I met someone like me!”: Autistic adolescents and their parent’s experience of the KONTAKT® social skills group training. Journal of Autism and Developmental Disorders.

[CR4] American Psychiatric Association (1994). Diagnostic and statistical manual of mental disorders.

[CR5] American Psychiatric Association (2013). *Diagnostic and statistical manual of mental disorders*, (5th ed.). American Psychiatric Association. 10.1176/appi.books.9780890425596

[CR6] Askari S, Anaby D, Bergthorson M, Majnemer A, Elsabbagh M, Zwaigenbaum L (2015). Participation of children and youth with autism spectrum disorder: A scoping review. Review Journal of Autism and Developmental Disorders.

[CR7] Australian Bureau of Statistics. (2019). *Disability, Ageing and Carers, Australia: Summary of Findings, 2018*. Retrieved from https://www.abs.gov.au/ausstats/abs@.nsf/lookup/4430.0main+features102018

[CR8] Baron-Cohen S, Wheelwright S (2004). The empathy quotient: An investigation of adults with Asperger syndrome or high functioning autism, and normal sex differences. Journal of Autism and Developmental Disorders.

[CR9] Barton EE, Fettig A (2013). Parent-implemented programs for young children with disabilities: A review of fidelity features. Journal of Early Program.

[CR10] Bartoš F, Maier M, Quintana DS, Wagenmakers E (2020). Adjusting for publication bias in JASP & R: Selection models, PET-PEESE, and Robust Bayesian meta-analysis. Advances in Methods and Practices in Psychological Science.

[CR11] Bauminger N (2002). The facilitation of social-emotional understanding and social interaction in high-functioning children with autism: Program outcomes. Journal of Autism and Developmental Disorders.

[CR12] Bauminger N, Kasari C (2003). Loneliness and friendship in high-functioning children with autism. Child Development.

[CR13] Bauminger N, Shulman C (2003). The development and maintenance of friendship in high-functioning children with autism: Maternal perceptions. Autism.

[CR14] Bellg AJ, Borrelli B, Resnick B, Hecht J, Minicucci DS, Ory M, Ogedegbe G, Orwig D, Ernst D, Czajkowski S (2004). Enhancing treatment fidelity in health behavior change studies: Best practices and recommendations from the NIH Behavior Change Consortium. Health Psychology.

[CR127] Björling Elin A., Thomas Kyle, Rose Emma J., Cakmak Maya (2020). Exploring teens as robot operators, users and witnesses in the wild. Frontiers in Robotics and AI.

[CR130] Bölte, S. (2018).* Social färdighetsträning i grupp med fokus på kommunikation och social interaktion vid autismspektrumtillstånd enligt Frankfurtmodellen (KONTAKT).* HOGFREFE.

[CR15] Bölte S, Lawson WB, Marschik PB, Girdler S (2021). Reconciling the seemingly irreconcilable: The WHO's ICF system integrates biological and psychosocial environmental determinants of autism and ADHD. BioEssays.

[CR16] Borenstein M, Hedges LV, Higgins JPT, Rothstein HR, Borenstein M, Hedges LV, Higgins JPT, Rothstein HR (2009). Multiple outcomes or time points within a study. Introduction to meta-analysis.

[CR18] Borrelli B (2011). The assessment, monitoring, and enhancement of treatment fidelity in public health clinical trials. Journal of Public Health Dentistry.

[CR19] Borrelli B, Sepinwall D, Ernst D, Bellg AJ, Czajkowski S, Breger R, DeFrancesco C, Levesque C, Sharp DL, Ogedegbe G, Resnick B, Orwig D (2005). A new tool to assess treatment fidelity and evaluation of treatment fidelity across 10 years of health behavior research. Journal of Consulting and Clinical Psychology.

[CR124] Bottema-Beutel K., Kapp S.K., Lester J.N., Sasson N.J., Hand N. (2021). Avoiding ableist language: Suggestions for autism researchers. Autism in Adulthood.

[CR20] Boutron I, Moher D, Altman DG, Schulz KF, Ravaud P (2008). Extending the CONSORT statement to randomized trials of nonpharmacologic treatment: Explanation and elaboration. Annals of Internal Medicine.

[CR21] Bukowski W, Hoza B, Boivin M (1994). Measuring friendship quality during pre and early adolescence: The development and psychometric properties of the Friendship Qualities Scale. Journal of Social and Personal Relationships.

[CR22] Bukowski W, Newcomb A, Hartup W (1996). The company they keep: Friendship in childhood and adolescence.

[CR23] Cage E, Di Monaco J, Newell V (2018). Experiences of autism acceptance and mental health in autistic adults. Journal of Autism and Developmental Disorders.

[CR25] Cassidy S, Bradley L, Shaw R, Baron-Cohen S (2018). Risk markers for suicidality in autistic adults. Molecular Autism.

[CR24] Cassidy SA, Gould K, Townsend E, Pelton M, Robertson AE, Rodgers J (2020). Is camouflaging autistic traits associated with suicidal thoughts and behaviours? Expanding the interpersonal psychological theory of suicide in an undergraduate student sample. Journal of Autism and Developmental Disorders.

[CR26] Chevallier C, Kohls G, Troiani V, Brodkin ES, Schultz RT (2012). The social motivation theory of autism. Trends in Cognitive Sciences.

[CR27] Cholemkery H, Freitag CM (2014). SOSTA-FRA: Soziales Kompetenztraining fur Kinder und Jugendliche mit Autismus- Spektrum-Störungen.

[CR29] *Choque Olsson, N., Flygare, O., Coco, C., Görling, A., Råde, A., Chen, Q., Lindstedt, K., Berggren, S., Serlachius, E., Jonsson, U., Tammimies, K., Kjellin, L., & Bölte, S. (2017). Social skills training for children and adolescents with autism spectrum disorder: A randomized controlled trial. *Journal of American Child and Adolescent Psychiatry, 56*(7), 585–592.10.1016/j.jaac.2017.05.001 10.1016/j.jaac.2017.05.001 28647010

[CR30] Choque Olsson N, Rautio D, Asztalos J, Stoetzer U, Bölte S (2016). Social skills group training in high-functioning autism: A qualitative responder study. Autism.

[CR32] Constantino J, Gruber C (2005). Social responsiveness Scale (SRS): Technical Manual.

[CR33] Constantino J, Gruber C (2012). SRS-2: Screening for autism spectrum disorder.

[CR34] Corbett BA, Gunther JR, Comins D, Price J, Ryan N, Simon D (2011). Brief report: Theatre as therapy for children with autism spectrum disorder. Journal of Autism and Developmental Disorders.

[CR35] *Corbett, B. A., Ioannou, S., Key, A. P., Coke, C., Muscatello, R., Vandekar, S., & Muse, I. (2019). Treatment effects in social cognition and behavior following a theatre-based program for youth with autism. *Developmental Neuropsychology, 44*(7), 481–494.10.1080/87565641.2019.1676244 10.1080/87565641.2019.1676244 PMC6818093 31589087

[CR36] *Corbett, B. A., Key, A. P., Qualls, L., Fecteau, S., Newsom, C., Coke, C., & Yoder, P. (2016). Improvement in social competence using a randomized trial of a theatre program for children with autism spectrum disorder. *Journal of Autism and Developmental Disorders, 46*(2), 658–672.10.1007/s10803-015-2600-9 10.1007/s10803-015-2600-9 PMC5633031 26419766

[CR37] Craig P, Dieppe P, Macintyre S, Michie S, Nazareth I, Petticrew M (2008). Developing and evaluating complex programs: The new Medical Research Council guidance. British Medical Journal.

[CR38] Cridland EK, Jones SC, Caputi P, Magee CA (2014). Being a girl in a boys' world: Investigating the experiences of girls with autism spectrum disorders during adolescence. Journal of Autism and Developmental Disorders.

[CR39] Dalton JE, Bolen SD, Mascha EJ (2016). Publication bias: The elephant in the review. Anesthesia & Analgesia.

[CR40] Dean M, Harwood R, Kasari C (2017). The art of camouflage: Gender differences in the social behaviors of girls and boys with autism spectrum disorder. Autism.

[CR41] Del Re, A. (2013). *Compute.es: Compute effect sizes* (Version 0.2–2) [Computer software]. R package. https://www.acdelre.com

[CR42] Del Re AC (2015). A practical tutorial on conducting meta-analysis in R. The Quantitative Methods for Psychology.

[CR43] Del Re, A. C., & Hoyt, W. T. (2018). *MAd: Meta-analysis with mean differences* (Version 0.8) [Computer software]. R package. https://cran.r-project.org/web/packages/MAd/MAd.pdf

[CR45] DuBois D, Bull C, Sherman M, Roberts M (1998). Self-esteem and adjustment in early adolescence: A social-contextual perspective. Journal of Youth and Adolescence.

[CR47] Egger M, Davey SG, Martin S, Christoph M (1997). Bias in meta-analysis detected by a simple, graphical test. British Medical Journal.

[CR48] Enea M, Plaia A (2014). Influence Diagnostics for meta-analysis of individual patient data using generalized linear mixed models.

[CR49] Everitt BS, Wessely S (2008). Clinical trials in psychiatry.

[CR129] Fletcher-Watson S, Adams J, Brook K, Charman T, Crane L, Cusack J, Leekam S, Milton D, Parr JR, Pellicano E (2019). Making the future together: Shaping autism research through meaningful participation. Autism.

[CR52] Frankel, F., & Mintz, J. (2008). *Measuring the quality of play dates (QPQ): Technical manual*. UCLA Parenting and Children’s Friendship Program.

[CR53] *Freitag, C. M., Jensen, K., Elsuni, L., Sachse, M., Herpertz-Dahlmann, B., Schulte-Ruther, M., Hanig, S., Gontard, A., Poustka, L., Schad-Hansjosten, T., Wenzl, C., Sinzig, J., Taurines, R., Geisler, J., Kieser, M., & Cholemkery, H. (2016). Group-based cognitive behavioural psychotherapy for children and adolescents with ASD: The randomized, multicentre, controlled SOSTA-net trial. *Journal of Child Psychology and Psychiatry, 57*(5), 596–605.10.1111/jcpp.12509 10.1111/jcpp.12509 26715086

[CR54] Fritz CO, Morris PE, Richler JJ (2012). Effect size estimates: Current use, calculations, and interpretation. Journal of Experimental Psychology: General.

[CR55] Gates J, Kang E, Lerner M (2017). Efficacy of group social skills programs for youth with autism spectrum disorder: A systematic review and meta-analysis. Clinical Psychology Review.

[CR126] Gillespie-Lynch K, Bublitz D, Donachie A, Wong V, Brooks PJ, D’Onofrio J (2017). “For a Long Time Our Voices have been Hushed”: Using Student Perspectives to Develop Supports for Neurodiverse College Students. Frontiers in Psychology.

[CR56] Gresham F, Elliott S (1990). The Social Skills Rating System (SSRS): Technical manual.

[CR57] Gresham F, Elliott S (2008). Social Skills Improvement System (SSIS) rating scales: Technical manual.

[CR58] Guivarch J, Murdymootoo V, Elissalde SN, Salle-Collemiche X, Tardieu S, Jouve E, Poinso F (2017). Impact of an implicit social skills training group in children with autism spectrum disorder without intellectual disability: A before-and-after study. PLoS ONE.

[CR59] Gupta S (2011). Intention-to-treat concept: A review. Perspectives in Clinical Research.

[CR60] Harden SM, Gaglio B, Shoup JA, Kinney KA, Johnson SB, Brito F, Blackman KCA, Zoellner JM, Hill JL, Almeida FA, Glasgow RE, Estabrooks PA (2015). Fidelity to and comparative results across behavioral programs evaluated through the RE-AIM framework: A systematic review. Systematic Reviews.

[CR61] Hare TA, Tottenham N, Galvan A, Voss HU, Glover GH, Casey BJ (2008). Biological substrates of emotional reactivity and regulation in adolescence during an emotional Go-No go task. Biological Psychiatry.

[CR63] Harrison P, Boney T, Thomas A, Grimes J (2002). Best practices in adaptive behaviour assessment. Best practices in school psychology IV.

[CR64] Higgins, J. P. T., & Green, S. (2011). *Cochrane handbook for systematic reviews of programs*. The Cochrane Collaboration.

[CR65] Higgins JPT, Thompson SG, Deeks JJ, Altman DG (2003). Measuring inconsistency in meta-analyses. British Medical Journal.

[CR128] Hodgetts S, Richards K, Park E (2018). Preparing for the future: Multi-stakeholder perspectives on autonomous goal setting for adolescents with autism spectrum disorders. Disability and Rehabilitation.

[CR66] Howlin P, Magiati I (2017). Autism spectrum disorder: Outcomes in adulthood. Current Opinion in Psychology.

[CR67] Johnson-Kozlow M, Hovell MF, Rovniak LS, Sirikulvadhana L, Wahlgren DR, Zakarian JM (2008). Fidelity issues in secondhand smoking programs for children. Nicotine & Tobacco Research.

[CR69] Jonsson U, Choque Olsson N, Bölte S (2016). Can findings from randomized controlled trials of social skills training in autism spectrum disorder be generalized? The neglected dimension of external validity. Autism: the International Journal of Research and Practice.

[CR68] *Jonsson, U., Choque Olsson, N. C., Coco, C., Görling, A., Flygare, O., Råde, A., Chen, Q., Berggren, S., Tammimies, K., & Bölte, S. (2018). Long-term social skills group training for children and adolescents with autism spectrum disorder: A randomized controlled trial. *European Child & Adolescent Psychiatry, 28*(2), 189–201.10.1007/s00787-018-1161-9 10.1007/s00787-018-1161-9 PMC6510850 29748736

[CR70] Kang E, Gioia A, Pugliese CE, Islam NY, Martinez-Pedraza FL, Girard RM, McLeod BD, Carter AS, Lerner M (2021). Alliance-outcome associations in a community-based social skills program for youth with autism spectrum disorder. Behavior Therapy.

[CR71] Karlsson P, Bergmark A (2015). Compared with what? An analysis of control-group types in Cochrane and Campbell reviews of psychosocial treatment efficacy with substance use disorders. Addiction.

[CR72] Kasari C, Dean M, Kretzmann M, Shih W, Orlich F, Whitney R, Landa R, Lord C, King B (2016). Children with autism spectrum disorder and social skills groups at school: A randomized trial comparing program approach and peer composition. Journal of Child Psychology and Psychiatry.

[CR74] Kenny L, Hattersley C, Molins B, Buckley C, Povey C, Pellicano E (2016). Which terms should be used to describe autism? Perspectives from the UK autism community. Autism.

[CR75] Kiresuk TJ, Smith A, Cardillo JE (1994). Goal Attainment Scaling: applications, theory, and measurement.

[CR76] Kmet LM, Cook LS, Lee RC (2004). Standard quality assessment criteria for evaluating primary research papers from a variety of fields. Heritage Foundation for Medical Research.

[CR77] *Ko, J. A., Miller, A. R., & Vernon, T. W. (2019). Social conversation skill improvements associated with the Social Tools And Rules for Teens program for adolescents with autism spectrum disorder: Results of a randomized controlled trial*. Autism, 23*(5), 1224–1235.10.1177/1362361318808781 10.1177/1362361318808781 30378448

[CR78] Korkman, M., Kirk, U., & Kemp, S. (2007). *NEPSY: Technical manual*. Harcourt Assessment.

[CR79] Laugeson E, Frankel F (2010). Social skills for teenagers with developmental and autism spectrum disorders: The PEERS® treatment manual.

[CR80] *Laugeson, E., Frankel, F., Mogil, C., & Dillon, A. R. (2009). Parent-assisted social skills training to improve friendships in teens with autism spectrum disorders. Journal of Autism & Developmental Disorders, 39(4), 596–606. 10.1007/s10803-008-0664-5 10.1007/s10803-008-0664-5 19015968

[CR81] Lee L, Packer TL, Tang SH, Girdler S (2008). Self-management education programs for age-related macular degeneration: A systematic review. Australasian Journal on Ageing.

[CR82] *Lerner, M., & Mikami, A. (2012). A preliminary randomized controlled trial of two social skills programs for youth with high-functioning autism spectrum disorders. *Focus on Autism & Other Developmental Disabilities, 27*(3), 147–157. 10.1177/1088357612450613

[CR83] Lerner M, White WS, McPartland JC (2012). Mechanisms of change in psychosocial programs for autism spectrum disorders. Dialogues in Clinical Neuroscience.

[CR84] Liberati A, Altman DG, Tetzlaff J, Mulrow C, Gøtzsche PC, Ioannidis J (2009). The PRISMA statement for reporting systematic reviews and meta-analyses of studies that evaluate healthcare programs: Explanation and elaboration. British Journal of Medicine.

[CR85] Lord C, Charman T, Havdahl A, Carbone P, Anagnostou E, Boyd B, Carr T, de Vries PJ, Dissanayake C, Divan G, Freitag CM, Gotelli MM, Kasari C, Knapp M, Mundy P, Plank A, Scahill L, Servili C, Shattuck P (2021). The Lancet Commission on the future of care and clinical research in autism. Lancet.

[CR88] Lurie J, Morgan T (2013). Pros and cons of pragmatic clinical trials. Journal of Comparative Effectiveness Research.

[CR89] Majnemer A, Shikako-Thomas K, Schmitz N, Shevell M, Lach L (2015). Stability of leisure participation from school-age to adolescence in individuals with cerebral palsy. Research in Developmental Disabilities.

[CR90] Mandell DS, Stahmer AC, Shin S, Xie M, Reisinger E, Marcus SC, Mandell D (2013). The role of treatment fidelity on outcomes during a randomized field trial of an autism program. Autism.

[CR91] Marsiglia FF, Booth JM (2015). Cultural adaptation of interventions in real practice settings. Research on Social Work Practice.

[CR92] *Matthews, N. L., Laflin, J., Orr, B. C., Warriner, K., DeCarlo, M., & Smith, C. J. (2020). Brief report: Effectiveness of an accelerated version of the PEERS® social skills program for adolescents. *Journal of Autism and Developmental Disorders, 50*(6), 2201–2207. 10.1007/s10803-019-03939-9 10.1007/s10803-019-03939-9 30825083

[CR93] *Matthews, N. L., Orr, B. C., Warriner, K., DeCarlo, M., Sorensen, M., Laflin, J., & Smith, C. J. (2018). Exploring the effectiveness of a peer-mediated model of the PEERS® curriculum: A pilot randomized control trial. *Journal of Autism and Developmental Disorders, 48*(7), 2458–2475.10.1007/s10803-018-3504-2 10.1007/s10803-018-3504-2 29453708

[CR94] Mattick R, Clarke C (1998). Development and validation of measures of social phobia scrutiny fear and social interaction anxiety. Behaviour Research and Therapy.

[CR95] McMahon C, Lerner M, Britton N (2013). Group-based social skills programs for adolescents with higher-functioning autism spectrum disorder: A review and looking to the future. Adolescent Health, Medicine and Therapeutics.

[CR96] Moher D, Hopewell S, Schulz K, Montori V, Gøtzsche P, Devereaux P, Elbourne D, Egger M, Altman D (2010). CONSORT 2010 explanation and elaboration: Updated guidelines for reporting parallel group randomised trials. British Medical Journal.

[CR97] Monahan J, Freedman B, Pini K, Lloyd R (2021). Autistic input in social skills interventions for young adults: A systematic review of the literature. Review Journal of Autism and Developmental Disorders.

[CR98] Morris S (2008). Estimating effect sizes from pretest-posttest control group designs. Organizational Research Methods.

[CR99] Naveed S, Waqas A, Amray AN, Memon RI, Javed N, Tahir MA, Ghozy S, Jahan N, Khan AS, Rahman A (2019). Implementation and effectiveness of non-specialist mediated programs for children with autism spectrum disorder: A systematic review and meta-analysis. PLoS ONE.

[CR100] *Rabin, S. J., Israel-Yaacov, S., Laugeson, E. A., Mor-Snir, I., & Golan, O. A. (2018). A randomized controlled trial evaluating the Hebrew adaptation of the PEERS® program: Behavioral and questionnaire-based outcomes. *Autism Research, 11*(8), 1187–1200.10.1002/aur.1974 10.1002/aur.1974 30095232

[CR101] Radley KC, Dart EH, Brennan KJ, Helbig KA, Lehman EL, Silberman M, Mendanhall K (2020). Social skills teaching for individuals with autism spectrum disorder: A Systematic Review. Advances in Neurodevelopmental Disorders.

[CR102] Ratcliff K, Hong I, Hilton C (2018). Leisure participation patterns for school-age youth with autism spectrum disorders: Findings from the 2016 national survey of children’s health. Journal of Autism and Developmental Disorders.

[CR103] Ratto AB, Turner-Brown L, Rupp BM, Mesibov GB, Penn DL (2011). Development of the Contextual Assessment of Social Skills (CASS): A role-play measure of social skill for individuals with high functioning autism. Journal of Autism and Developmental Disorders.

[CR104] Reichow B, Steiner AM, Volkmar F (2010). Social skills groups for people aged 6 to 21 with autism spectrum disorders (ASD). Cochrane Database for Systematic Reviews.

[CR105] Rothenberger A, Becker A, Erhart M, Wille N, Ravens-Sieberer U (2008). Psychometric properties of the parent strengths and difficulties questionnaire in the general population of German children and adolescents: Results of the BELLA study. European Child & Adolescent Psychiatry.

[CR106] RStudio Team. (2015). *RStudio: Integrated development environment for R* [Computer software]. http://www.rstudio.com/

[CR107] Santacroce SJ, Maccarelli LM, Grey M (2004). Intervention fidelity. Nursing Research.

[CR108] *Schohl, K., Van Hecke, A., Carson, A., Dolan, B., Karst, J., & Stevens, S. S. (2014). A replication and extension of the PEERS® program: Examining effects on social skills and social anxiety in adolescents with autism spectrum disorders—program for the education and enrichment of relational skills. *Journal of Autism & Developmental Disorders, 44*(3), 532–545.10.1007/s10803-013-1900-1 10.1007/s10803-013-1900-1 23893101

[CR109] *Shum, K. K., Cho, W. K., Lam, L. M. O., Laugeson, E.A., Wong, W.S., & Law, L. S. K. (2019). Learning how to make friends for Chinese adolescents with autism spectrum disorder: A randomized controlled trial of the Hong Kong Chinese version of the PEERS® program. *Journal of Autism and Developmental Disorders, 49*(2), 527–541. 10.1007/s10803-018-3728-1 10.1007/s10803-018-3728-1 30143950

[CR110] Smetana J, Campione-Barr N, Metzger A (2006). Adolescent development in interpersonal and societal contexts. Annual Review of Psychology.

[CR111] Smith T, Scahill L, Dawson G, Guthrie D, Lord C, Odom S, Rogers S, Wagner A (2007). Designing research studies on psychosocial programs in autism. Journal of Autism and Developmental Disorders.

[CR113] Tseng A, Biagianti B, Francis SM, Conelea CA, Jacob S (2020). Social cognitive programs for adolescents with autism spectrum disorders: A systematic review. Journal of Affective Disorders.

[CR114] *Van Hecke, A. V., Stevens, S., Carson, A. M., Karst, J. S., Dolan, B., Schohl, K., McKindles, R. J., Remmel, R., & Brockman, S. (2015). Measuring the plasticity of social approach: A randomized controlled trial of the effects of the PEERS® program on EEG asymmetry in adolescents with autism spectrum disorders*. Journal of Autism and Developmental Disorders, 45*(2), 316–335.10.1007/s10803-013-1883-y 10.1007/s10803-013-1883-y 23812665

[CR116] *Vernon, T. W., Miller, A. R., Ko, J. A., Barrett, A. C., & McGarry, E. S. (2018). A randomized controlled trial of the Social Tools And Rules for Teens (START) program: An immersive socialization program for adolescents with autism spectrum disorder. *Journal of autism and developmental disorders, 48*(3), 892–904. 10.1007/s10803-017-3380-1 10.1007/s10803-017-3380-1 29164444

[CR115] Vernon TW, Miller AR, Ko JA, Wu V (2016). Social Tools And Rules for Teens (The START program): Program description and preliminary outcomes of a multi-component socialization program for adolescents with autism spectrum disorder. Journal of Autism and Developmental Disorders.

[CR118] Wells M, Williams B, Treweek S, Coyle J, Taylor J (2012). Program description is not enough: Evidence from an in-depth multiple case study on the untold role and impact of context in randomised controlled trials of seven complex programs. Trials.

[CR120] White SW, Albano A, Johnson C, Kasari C, Ollendick T, Klin A (2010). Development of a cognitive-behavioral program to treat anxiety and social deficits in teens with high-functioning autism. Clinical Child and Family Psychology Review.

[CR119] *White, S. W., Ollendick, T., Albano, A. M., Oswald, D., Johnson, C. R., Southam-Gerow, M. A., Kim, I., & Scahill, L. (2013). Randomized controlled trial: Multimodal anxiety and social skill program for adolescents with autism spectrum disorder*. Journal of Autism and Developmental Disorders, 43*(2), 382–394. 10.1007/s10803-012-1577-x 10.1007/s10803-012-1577-x PMC3494811 22735897

[CR121] Williams JB, Popp D, Kobak KA, Detke MJ (2012). The power of expectation bias. European Psychiatry.

[CR122] Wolstencroft J, Robinson L, Srinivasan R, Kerry E, Mandy W, Skuse D (2018). A systematic review of group social skills programs, and meta-analysis of outcomes, for children with high functioning ASD. Journal of Autism and Developmental Disorders.

[CR123] *Yoo, H. J., Bahn, G., Cho, I. H., Kim, E. K., Kim, J. H., Min, J. W., Lee, W. H., Seo, J. S., Jun, S. S., Bong, G., Cho, S., Shin, M. S., Kim, B. N., Kim, J. W., Park, S., & Laugeson, E. A. (2014). A randomized controlled trial of the Korean version of the PEERS® parent-assisted social skills training program for teens with ASD. *Autism Research, 7*(1), 145–161. 10.1002/aur.1354 10.1002/aur.1354 24408892

